# miR156‐independent repression of the ageing pathway by longevity‐promoting AHL proteins in Arabidopsis

**DOI:** 10.1111/nph.18292

**Published:** 2022-06-26

**Authors:** Arezoo Rahimi, Omid Karami, Salma Balazadeh, Remko Offringa

**Affiliations:** ^1^ Plant Developmental Genetics, Institute of Biology Leiden Leiden University Sylviusweg 72 2333 BE Leiden the Netherlands; ^2^ Plant Molecular Stress Biology, Institute of Biology Leiden Leiden University Sylviusweg 72 2333 BE Leiden the Netherlands

**Keywords:** *AHL* genes, *Arabidopsis thaliana* (Arabidopsis), flowering, heteroblastic leaf development, *miR156/miR157*, reproductive phase change, *SPL* genes, vegetative phase change

## Abstract

- Plants age by developmental phase changes. In Arabidopsis, the juvenile to adult vegetative phase change (VPC) is marked by clear heteroblastic changes in leaves. VPC and the subsequent vegetative to reproductive phase change are promoted by SQUAMOSA PROMOTOR BINDING PROTEIN‐LIKE (SPL) transcription factors and repressed by miR156/157 targeting *SPL* transcripts.
- By genetic, phenotypic, and gene expression analyses, we studied the role of the longevity‐promoting AT‐HOOK MOTIF NUCLEAR LOCALIZED 15 (AHL15) and family members in SPL‐driven plant ageing.
- Arabidopsis *ahl* loss‐of‐function mutants showed accelerated VPC and flowering, whereas *AHL15* overexpression delayed these phase changes. Expression analysis and tissue‐specific *AHL15* overexpression revealed that AHL15 affects VPC and flowering time directly through its expression in the shoot apical meristem and young leaves, and that AHL15 represses *SPL2/9/13/15* gene expression in a miR156/157‐independent manner. The juvenile traits of *spl* loss‐of‐function mutants appeared to depend on enhanced expression of the *AHL15* gene, whereas SPL activity prevented vegetative growth from axillary meristem by repressing *AHL15* expression.
- Our results place AHL15 and close family members together with SPLs in a reciprocal regulatory feedback loop that modulates VPC, flowering time, and axillary meristem development in response to both internal and external signals.

## Introduction

Plant development progresses through several distinct developmental phases, starting with embryogenesis and followed successively by the juvenile vegetative, adult vegetative, reproductive, and gametophytic phases. In the juvenile vegetative phase, plants are generally not competent to flower, and flowering requires the transition from juvenile to adult vegetative development, which is referred to as vegetative phase change (VPC). Depending on the species, VPC may be marked by morphological changes, such as increased internode length, adventitious root production, and changes in leaf size and shape and trichome distribution, resulting in the presence of both juvenile and adult organs on a plant, a phenomenon referred to as heteroblasty (Huijser & Schmid, 2011). In *Arabidopsis thaliana* (Arabidopsis), leaf heteroblasty provides a clear indicator of VPC. Juvenile leaves have smooth margins, are rounder (length : width), and lack abaxial trichomes, whereas adult leaves have serrated margins, are more elongated (length : width ratio) and have abaxial trichomes (Telfer *et al*., 1997).

Compared with the adult‐to‐reproductive phase transition (or reproductive phase change), which is one of the key traits in crops, much less is known about the molecular mechanisms that mediate VPC. However, research in Arabidopsis has demonstrated that microRNAs (miRNAs) miR156, miR157, and miR172, are major regulators of VPC in Arabidopsis and other plant species (Poethig, 2013; Teotia & Tang, 2015). During the Arabidopsis life cycle the gradual decrease in *miR156/miR157* expression results in increased expression of the *SQUAMOSA‐PROMOTER BINDING PROTEIN‐LIKE* (*SPL*) *miR156*/*miR157* target genes. SPL transcription factors, in turn, promote the adult developmental programme at the shoot apical meristem (SAM), resulting in the transition from juvenile to adult leaf production and eventually from vegetative to reproductive development (Wu *et al*., 2009; He *et al*., 2018). The gradual decrease of *miR156*/*miR157* expression during shoot maturation is accompanied by an SPL‐induced increase in *miR172* expression. miR172 promotes the development of trichomes on the abaxial side of leaves by repressing the expression of the APETALA2‐LIKE (AP2‐like) transcription factors TARGET OF EARLY ACTIVATION TAGGED1 (TOE1) and TOE2 (Wu *et al*., 2009; Wang *et al*., 2019; Xu *et al*., 2019). In addition, SPLs promote the other adult leaf traits, such as leaf elongation and leaf serration, independent of miR172 (Wu *et al*., 2009). However, the genes that act downstream of SPLs and TOE1/TOE2 in VPC remain unidentified.

In eukaryotes, a wide range of DNA binding proteins have been identified that bind to AT‐rich sequences in the minor groove of DNA by a small AT‐hook domain (Reeves, 2010). These AT‐hook proteins are considered as chromatin architectural factors involved in a diverse array of crucial cellular processes, including cell growth, cell differentiation, cell transformation, cell proliferation, cell death, and DNA replication and repair, by regulating chromatin remodelling and gene transcription (Reeves, 2010; Sgarra *et al*., 2010; Ozturk *et al*., 2014). The Arabidopsis genome encodes 29 AT‐HOOK MOTIF NUCLEAR‐LOCALIZED (AHL) proteins that contain either one or two AT‐hook domains and a Plant and Prokaryote Conserved (PPC) domain (Fujimoto *et al*., 2004; Matsushita *et al*., 2007; Street *et al*., 2008; Zhao *et al*., 2013). These AHL proteins have been shown to be implicated in several aspects of plant growth and development, including hypocotyl growth (Street *et al*., 2008; Xiao *et al*., 2009; Zhao *et al*., 2013), root vascular tissue differentiation (Zhou *et al*., 2013), flower development (Ng *et al*., 2009), and flowering time (Street *et al*., 2008; Xiao *et al*., 2009; Yun *et al*., 2012; Xu *et al*., 2013). Based on mutant and protein–protein interaction studies, the AHL family members have been proposed to bind AT‐rich DNA regions as hetero‐multimeric complexes that recruit other transcription factors through their interacting PPC domains (Zhao *et al*., 2013). In addition, AHL proteins have been shown to repress transcription of several key developmental regulatory genes, possibly through modulation of the epigenetic code in the vicinity of their DNA binding regions (Lim *et al*., 2007; Ng *et al*., 2009; Yun *et al*., 2012). Some evidence has been obtained that AHL proteins function by altering the organization of chromatin structure (Lim *et al*., 2007; Ng *et al*., 2009; Yun *et al*., 2012; Xu *et al*., 2013). However, since this plant‐specific class of nuclear proteins has only been studied more recently, their exact mode of action is still elusive.

Recently, we have shown that the *AHL15* gene and other members of the *AHL* gene family are important for embryogenesis (Karami *et al*., 2021) and that they promote plant longevity by delaying axillary meristem (AM) maturation (Karami *et al*., 2020). In view of the seemingly antagonistic effect of *AHL15* and its family members on the plant ageing pathway, we here study their possible role in the regulation of VPC and flowering time. Our analyses in Arabidopsis and *Nicotiana tabacum* (tobacco) showed that *AHL15* overexpression prolongs the juvenile vegetative phase and delays flowering, whereas *ahl15* loss of function results in precocious appearance of adult vegetative traits. A more detailed analysis indicated that AHL15 delays developmental phase changes by repressing *SPL* gene expression in an miRNA‐independent manner. We further show that, in turn, *AHL15* expression is repressed through feedback regulation by the SPLs.

## Materials and Methods

### Plant material and growth conditions and phenotype analysis

All Arabidopsis mutant and transgenic lines used in this study are in the Columbia (Col‐0) background. The *ahl15*, *ahl19*, *p35S:AHL15*, *p35S:AHL15‐GR*, *pAHL15:GUS*, *pAHL15:AHL15*, and *pAHL15:AHL15‐ΔG*, *p35S:amiRAHL20* and *ahl15/+ pAHL15:AHL15‐ΔG*, *pAHL15:AHL15‐GUS*, and *ahl15/+ pAHL15:AHL15‐GUS* plant lines have been described previously (Karami *et al*., 2020, 2021). The *spl9 spl15*, *p35S:miR156*, *p35S:MIM156*, *pSPL9:rSPL9‐GR*, *pSPL2:rSPL2‐GUS*, *pSPL9:rSPL9‐GUS*, *pSPL10:rSPL10‐GUS, pSPL11:rSPL11‐GUS*, *pSPL13:rSPL13‐GUS*, *pSPL15:rSPL15‐GUS*, *mirR156a/b*, *mirR156a/b/d miR157a/c*, and *spl2/9/10/11/13/15* plant lines were obtained from the Nottingham Arabidopsis Stock Centre. The reporter lines *pmiR156A:GUS*, *pmiR156B:GUS*, and *pmiR156C:GUS* have been described previously (Yu *et al*., 2015). Plant lines and F_1_, F_2_, or F_3_ plants from crosses were PCR genotyped using primers described in the Supporting Information Table S1. Seeds were directly sown on soil in pots and grown at 21°C, 65% relative humidity, and a 16 h (long day, LD) or 8 h (short day, SD) photoperiod. One exception: SD was 10 h for the top panel of Fig. S3 (see later). To score for aerial rosette leaf production by AMs, wild‐type, mutant, or transgenic plants were transferred to larger pots about 2 wk after flowering. *Nicotiana tabacum* cv SR1 Petit Havana (tobacco) wild‐type or *p35S:AHL15‐GR* plants (Karami *et al*., 2020) were grown in medium‐sized pots at 25°C, 70% relative humidity, and a 16 h photoperiod. For dexamethasone (DEX; Sigma‐Aldrich) treatment, Arabidopsis and tobacco plants were sprayed with 20 and 30 μM DEX, respectively. Leaf size (leaf length, and leaf width) was measured directly using a ruler.

The number of juvenile leaves (without abaxial trichomes) was scored once they appeared using a stereomicroscope. For imaging of leaf shape, fully expanded leaves were removed, attached to cardboard with double‐sided tape, flattened, and photographed with a Nikon D5300 camera (Nikon Corp., Tokyo, Japan). Leaf images were optimized and changed into black‐and‐white images and assembled using Adobe Illustrator CC 2017 (Adobe Inc., San Jose, CA, USA). Potted plants were photographed with a Nikon D5300 camera. All measurements were statistically analysed and plotted into graphs in GraphPad Prism 8 (GraphPad Software Inc., San Diego, CA, USA).

### Plasmid construction and transgenic Arabidopsis lines

To generate the constructs *pFD:AHL15* and *pANT:AHL15*, 3 kb regions upstream of the ATG initiation codon of the *FD* (AT4G35900) and *ANT* (AT4G37750) genes were amplified from Col‐0 genomic DNA using the forward and reverse PCR primers indicated in Table S1. The resulting fragments were first inserted into pDONR207 by BP reaction, and subsequently cloned upstream of the genomic fragment containing the *AHL15* transcribed region in destination vector *pGW‐AHL15* (Karami *et al*., 2021) by LR reaction. To generate *pFD:miR156*, first *pGW‐miR156* was constructed by replacing the *AHL15* fragment in *pGW‐AHL15* for a *Kpn*I and *Spe*I fragment containing the *miR156* transcribed region. The *FD* promoter fragment was subsequently recombined from pDONR207 into the resulting *pGW‐miR156* construct by LR reaction. All binary vectors were introduced into *Agrobacterium tumefaciens* strain AGL1 by electroporation (den Dulk‐Ras & Hooykaas, 1995), and Arabidopsis Col‐0 and *ahl15* plants were transformed using the floral dip method (Clough & Bent, 1998). The resulting plant lines and F_1_, F_2_, or F_3_ plants from crosses were PCR genotyped using primers described in the Table S1.

### Histochemical staining and microscopy

Histochemical staining of plant tissues for β‐glucuronidase (GUS) activity was performed using 1 mg l^−1^ X‐gluc (R0852; Thermo Fischer Scientific, Waltham, MA, USA) as described previously (Anandalakshmi *et al*., 1998). Tissues were stained for 4 h at 37°C, followed by chlorophyll extraction and rehydration by incubation for 10 min in a graded ethanol series (75%, 50%, and 25%). GUS‐stained tissues were observed and photographed using a Leica MZ12 microscope (Leica Geosystems AG, Heerbrugg, Switzerland) equipped with a Leica DC500 camera.

The tissue‐specific GUS‐staining intensity was quantified as mean grey values by analysing images of independent samples capturing the same region of interest using ImageJ, as previously described (Béziat *et al*., 2017).

### Quantitative real‐time PCR analysis

RNA was isolated from the shoot apex and a few young leaves close to the shoot apex (Fig. S1) or from the rosette base nodes using the RNEasy^©^ kit (Qiagen). First‐strand complementary DNA was synthesized using the RevertAid RT Reverse Transcription kit (Thermo Fisher Scientific). Quantitative PCR (qPCR) was performed on three biological replicates along with three technical replicates using the SYBR‐green dye premixed master‐mix (Thermo Fisher Scientific) in a C1000 Touch^©^ thermal cycler (Bio‐Rad, Hercules, CA, USA). *C*_t_ values were obtained using Bio‐Rad CFX Manager 3.1. The relative expression level of genes was calculated according to the $2^{-\Delta \Delta C_{t}}$ method (Livak & Schmittgen, 2001). The *β‐TUBULIN‐6* and *ACTIN2* genes were used as reference. Since similar results were obtained for these two genes in all qPCR experiments, expression was normalized using the *β‐TUBULIN‐6* gene. The miRNAs’ abundance was quantified using 1 μg of RNA in a reverse transcription reaction by using the stem–loop method with an miRNA‐specific forward primer. The miRNA levels were normalized using snoR101 as internal control. The data were analysed and plotted into graphs in GraphPad Prism 8. Three biological replicates were performed, with three technical replicates each. The primers used are described in Table S1.

## Results

### *AHL* genes delay vegetative phase change and flowering time

**Fig. 1:**
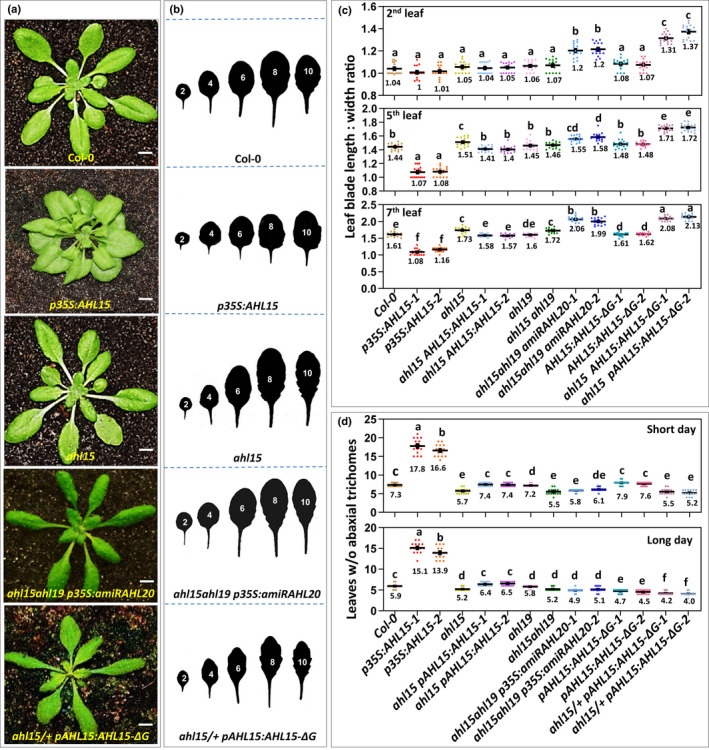
*AT‐HOOK MOTIF NUCLEAR LOCALIZED 15* (*AHL15*) and family members delay vegetative phase change in *Arabidopsis thaliana*. (a) The rosette phenotype of 5‐wk‐old wild‐type (Col‐0), *p35S:AHL15*, *ahl15*, *ahl15 ahl19 p35S:amiRAHL20*, or *ahl15/+ pAHL15:AHL15‐ΔG* plants grown under short day (SD) conditions. Bar, 1 cm. (b) Shape of the successive rosette leaves of 7‐wk‐old wild‐type, *p35S:AHL15*, *ahl15 ahl19 p35S:amiRAHL20*, or *ahl15/+ pAHL15:AHL15‐ΔG* plants grown under SD conditions. (c) The length : width ratio of the second, fifth, and seventh leaves of 7‐wk‐old wild‐type, *p35S:AHL15*, *ahl15*, *ahl15 pAHL15:AHL15*, *ahl19*, *ahl15 ahl19*, *ahl15 ahl19 p35S:amiRAHL20*, *pAHL15:AHL15‐ΔG*, and *ahl15/+ pAHL15:AHL15‐ΔG* plants grown under SD conditions. (d) The juvenile leaf number (leaves without abaxial trichomes) in wild‐type, *p35S:AHL15*, *ahl15*, *ahl15 pAHL15:AHL15*, *ahl19*, *ahl15 ahl19*, *ahl15 ahl19 p35S:amiRAHL20*, *pAHL15:AHL15‐ΔG*, and *ahl15/+ pAHL15:AHL15‐ΔG* plants grown under SD (up) and long day (down) conditions. (c, d) A coloured dot indicates the individual measurement per plant (*n* = 15 biologically independent plants per line), the horizontal line and the number below this line indicate the mean, and error bars indicate the SEM. Different letters indicate statistically significant differences (*P* < 0.01) as determined by one‐way ANOVA with Tukey's honest significant difference *post hoc* test.

When studying plants overexpressing *AHL15* (*p35S:AHL15*), we noticed that the leaf blade length : width ratio was significantly reduced compared with that of wild‐type plants (Fig. 1a–c). These initial observations and more detailed analysis showed that *p35S:AHL15* plants had a significantly increased number of leaves without abaxial trichomes, both under SD and LD conditions (Fig. 1d), indicating *AHL15* overexpression delayed VPC in Arabidopsis. By contrast, *ahl15* loss‐of‐function mutants developed leaves with a slightly increased blade length : width ratio and a reduced number of leaves without abaxial trichomes (Fig. 1a–d). *ahl15* mutant plants complemented with a *pAHL15:AHL15* genomic clone again showed wild‐type leaf development (Fig. 1a–d), indicating that the mutant phenotypes were caused by *ahl15* loss of function. *AHL15* is part of a large gene family in Arabidopsis, where it clusters together with two close homologues: *AHL19* and *AHL20* (Zhao *et al*., 2013; Karami *et al*., 2021). In line with the previously reported high degree of functional redundancy between *AHL* genes (Street *et al*., 2008; Xiao *et al*., 2009; Zhao *et al*., 2013; Karami *et al*., 2021), *ahl15 ahl19 p35S:amiRAHL20* triple‐mutant plants showed a stronger increase in the leaf blade length : width ratio compared with the *ahl15* single or *ahl15 ahl19* double mutants. However, the reduction in number of leaves without abaxial trichomes was comparable to that of *ahl15* or *ahl19* single or *ahl15 ahl19* double‐mutant plants under both SD and LD conditions (Fig. 1a–d).

**Fig. 2:**
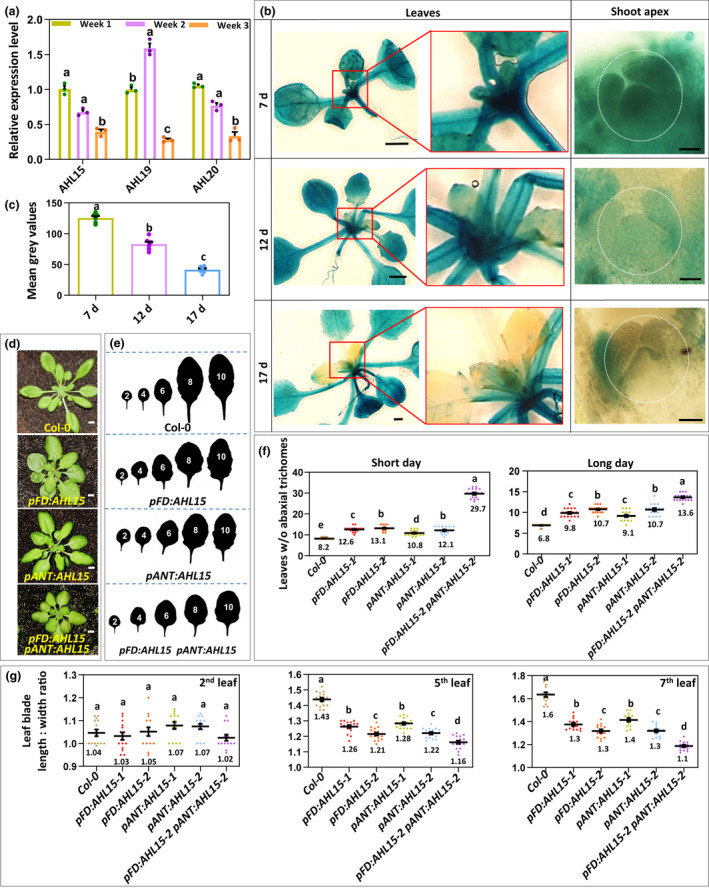
*AT‐HOOK MOTIF NUCLEAR LOCALIZED 15* (*AHL15*) expression in the shoot apex and young leaves delays vegetative phase change in *Arabidopsis thaliana*. (a) The relative expression level of *AHL15*, *AHL19*, and *AHL20* in the shoot apex and young leaves of 1‐, 2‑, and 3 wk‐old seedlings grown under short day (SD) conditions. Dots indicate the values of three biological replicates per plant line, bar indicates the mean, and error bars the SEM. Different letters indicate statistically significant differences (*P* < 0.05) as determined by a one‐way ANOVA with Tukey’s honest significant difference *post hoc* test. (b) Histochemical staining for and (c) quantification of β‐glucuronidase (GUS) activity in 7‑, 12‑, and 17‐d‐old *pAHL15:GUS* seedlings. Images in (b) show overview of leaves (left two rows) and details of the shoot apex (right row). The region indicated by the white dotted white line in (b, right row) was used for quantification in (c). (d) The rosette phenotype of 5‐wk‐old wild‐type (Col‐0), *pFD:AHL15*, *pANT:AHL15* and *pFD:AHL15 pANT:AHL15* plants grown under SD conditions. (e) Shape of successive rosette leaves of 7‐wk‐old of wild‐type, *pFD:AHL15*, *pANT:AHL15*, and *pFD:AHL15 pANT:AHL15* plants grown under SD conditions. (f) The juvenile leaf number (leaves without abaxial trichomes) in wild‐type, *pFD:AHL15*, *pANT:AHL15*, and *pFD:AHL15 pANT:AHL15* plants grown under SD and long day conditions. (h) The length : width ratio of the second, fifth, and seventh leaves of 7‐wk‐old wild‐type, *pFD:AHL15*, *pANT:AHL15*, and *pFD:AHL15 pANT:AHL15* plants grown under SD conditions. Coloured dots in graphs indicate the individual measurements (*n* = 6 biologically independent seedlings per line in (c) and *n* = 15 biologically independent plants per line in (f, g)), the horizontal line and the number below this line indicate the mean, and error bars indicate the SEM. Different letters indicate statistically significant differences (*P* < 0.01) as determined by one‐way ANOVA with Tukey’s honest significant difference *post hoc* test. Bars: (b, left panel, d) 1 cm; (b, right panel) 50 μm.

Previously, we and others have shown that it is possible to overcome the functional redundancy among *AHL* genes by expression of a dominant negative mutant version of an AHL protein. Such a mutant version can be obtained either by deleting the conserved GRFEIL motif in the PPC domain (ΔG) or by generating a C‐terminal fusion with GUS reporter protein (Zhao *et al*., 2013; Karami *et al*., 2020, 2021). Plants homozygous for a transgenic locus expressing AHL15‐ΔG under control of the *AHL15* promoter in the wild‐type background mostly showed wild‐type development, except for a decreased number of leaves without abaxial trichomes under LD conditions (Fig. 1c,d). By contrast, *pAHL15:AHL15‐GUS* plants showed an increase in the leaf blade length : width ratio and a decrease in the number of leaves without abaxial trichomes under, respectively, LD and SD conditions (Fig. S2a–d). Introduction of the *pAHL15:AHL15‐ΔG* or *pAHL15:AHL15‐GUS* loci into the *ahl15* mutant background by reciprocal crosses resulted in wild‐type F_1_ plants. F_2_ plants homozygous for both *ahl15* loss of function and the transgenic locus could not be obtained because of embryo lethality (Karami *et al*., 2020). However, selected *ahl15/+ pAHL15:AHL15‐ΔG* or *ahl15/+ pAHL15:AHL15‐GUS F2* plants, heterozygous for *ahl15* loss of function and homozygous for the transgenic locus, showed a strong increase in leaf blade length : width ratio and also a significant reduction in leaves without abaxial trichomes, indicative of early VPC (Figs 2a–d, S2a–d). Since Arabidopsis plants require VPC before they can shift to the reproductive phase (Huijser & Schmid, 2011), we also monitored the flowering time of our mutant lines, as quantified by the number of rosette leaves developed until flowering. The single or combined *ahl15* and *ahl19* loss‐of‐function mutations did not significantly affect flowering time, whereas *ahl15 ahl19 p35S:amiRAHL20* triple‐mutant plants showed a significant reduction in flowering time both under LD and SD conditions (Fig. S3a,b). By contrast, *p35S:AHL15* plants showed a significant delay of flowering under both SD and LD conditions (Fig. S3a,b). Moreover, previously, we already showed that *ahl15/+ pAHL15:AHL15‐ΔG* plants, but not *pAHL15:AHL15‐ΔG* plants, flowered significantly earlier under both LD and SD conditions compared with wild‐type plants (see also Fig. S3a). Here, we also observed a strong reduction in flowering time for both *pAHL15:AHL15‐GUS* and *ahl15/+ pAHL15:AHL15‐GUS* plants (Fig. S2e). Together, these results indicate that *AHL* genes act redundantly to delay VPC and, most likely as a result, also the reproductive phase change. Clearly, the AHL15‐GUS fusion has a stronger dominant negative effect on overcoming this redundancy than AHL15‐ΔG does.

We also analysed the effect of *AHL15* overexpression on the same developmental phase changes in tobacco, a plant species from a different family, using available *p35S:AHL15‐GR* tobacco lines (Karami *et al*., 2020). Previous analysis has shown that juvenile leaves in tobacco are rounder (lower length : width ratio) and smaller, show a reduced venation pattern, and are more pale green than adult leaves (Feng *et al*., 2016). In contrast to DEX‐treated wild‐type plants or mock‐treated *p35S:AHL15‐GR* tobacco plants, which showed wild‐type leaf development with three round juvenile leaves preceding the production of the typically larger and longer adult leaves (Feng *et al*., 2016), DEX‐treated *p35S:AHL15‐GR* plants formed many small leaves with juvenile features (round, pale green/yellow, reduced venation pattern; Fig. S4a). By spraying these plants once per week with DEX, the SAM continued to produce small leaves, and plants could be kept in the vegetative state for more than a year, resulting in highly branched and bushy plants that did not flower (Fig. S4b,c). By contrast, DEX‐treated wild‐type plants and *p35S:AHL15‐GR* control plants that were not treated with DEX developed normally and flowered after 2 months (not shown). This result indicates that *AHL15* overexpression can also strongly delay, if not prevent, VPC and flowering in a non‐Brassicaceae plant species, such as tobacco.

### *AHL15* delays vegetative phase change through its expression in the shoot apical meristem and in leaf primordia

To further understand the role of *AHL* genes in these developmental switches, we analysed the expression dynamics of *AHL15* and its close homologues *AHL19* and *AHL20* during VPC. Reverse transcription qPCR experiments showed that expression of all three *AHL* genes in the shoot apex and young leaves of 3‐wk‐old seedlings was reduced compared to 1‐wk‐old seedlings grown under short day (SD) conditions (Fig. 2a). The reduced *AHL15/19/20* expression in week 3 is in line with the timing of VPC, which occurs at 17–20 d after germination (DAG) in Arabidopsis ecotype Col‐0 grown under SD conditions (Xu *et al*., 2016). Similarly, analysis of *pAHL15:GUS* reporter lines showed that *AHL15* was expressed throughout the shoot at 7 DAG (week 1), that its expression declined in the SAM at 12 DAG (week 2), and that at 17 DAG (week 3) its expression was off in the SAM and severely reduced in the young leaves (Fig. 2b,c). The gene expression dynamics of *AHL15* and its close homologues supports their role in maintaining vegetative juvenile traits and thus suppressing VPC.

Previous studies have shown that VPC is regulated by both internal factors at the SAM (Fouracre & Poethig, 2019) and lateral organ‐derived signals (Yang *et al*., 2011, 2013; Yu *et al*., 2013). During VPC, *AHL15* remains expressed in expanded lateral organs, but its expression is reduced in the SAM and newly formed organs, suggesting that its expression in these tissues is most relevant for its function. To confirm this, we expressed *AHL15* under the control of the *FD* (*pFD*) and *AINTEGUMENTA* (*pANT*) promoters, which are predominantly active in the shoot apex (Yamaguchi *et al*., 2016; Fouracre & Poethig, 2019). Expression of *AHL15* under these promotors significantly delayed VPC, as indicated by the reduced length : width ratio of the leaves and increased number of leaves lacking abaxial trichomes (Fig. 2d–g), and also delayed flowering (Fig. S5a,b) under both SD and LD conditions. Combining both *pFD:AHL15* and *pANT:AHL15* constructs in one plant line led to a further delay of VPC (Fig. 2d–g) and flowering (Fig. S5a,b). This additive effect can be explained by the slightly different but overlapping activities of the selected promoters (Yamaguchi *et al*., 2016; Fouracre & Poethig, 2019), causing enhancement of *AHL15* expression in the SAM and in young leaf primordia (Fig. S6). Our results indicate that *AHL15* expression in these tissues is sufficient to delay VPC and flowering (Fig. 2b).

### AHL proteins repress *SPL* gene expression in an miR156/miR157‐independent manner

**Fig. 3:**
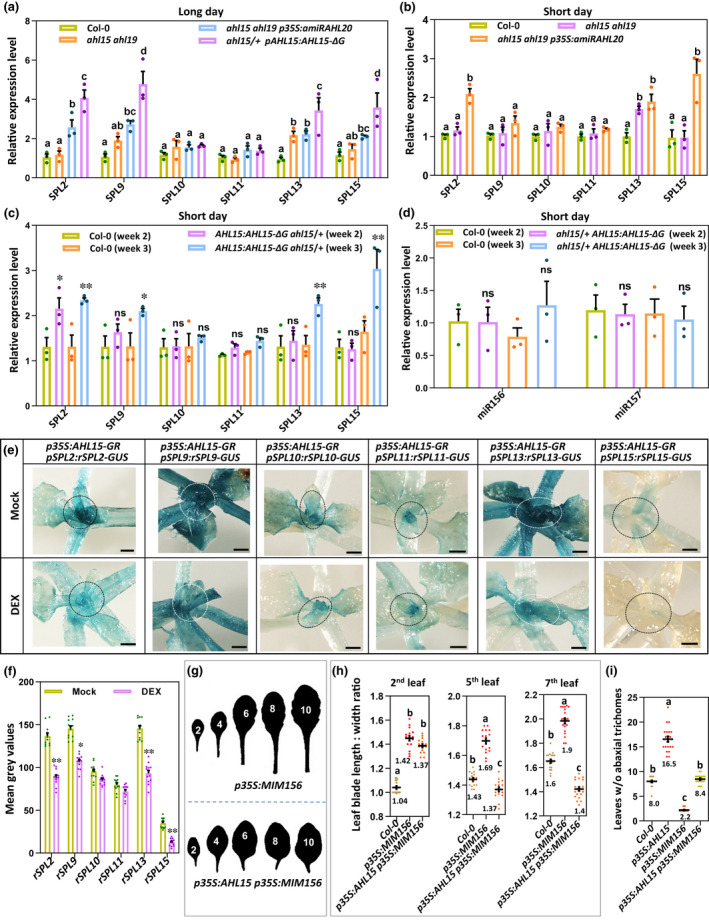
AT‐HOOK MOTIF NUCLEAR LOCALIZED 15 (AHL15) represses *SQUAMOSA PROMOTOR BINDING PROTEIN‐LIKE* (*SPL*) gene expression in a microRNA (miRNA)‐independent manner in *Arabidopsis thaliana*. (a, b) The relative expression level of *SPL2*, *SPL9*, *SPL10*, *SPL11*, *SPL13*, and *SPL15* in the shoot apex and young leaves of wild‐type, *ahl15 ahl19*, *ahl15 ahl19 p35S:amiRAHL20*, and *ahl15/+ pAHL15:AHL15‐ΔG* plants grown under (a) long day (LD) conditions for 10 d or (b) short day (SD) conditions for 3 wk. (c, d) The relative expression level of (c) *SPL2*, *SPL9*, *SPL10*, *SPL11*, *SPL13*, and *SPL15* or (d) *miR156* and *miR157* in the shoot apex and young leaves of 2‑ or 3‐wk‐old wild‐type or *ahl15/+ pAHL15:AHL15‐ΔG* plants grown under SD conditions. Dots in (a–d) indicate the values of three biological replicates per plant line, the bars indicate the mean, and error bars indicate the SEM. In (a, b), different letters indicate statistically significant differences (*P* < 0.05) as determined by a one‐way ANOVA with Tukey's honest significant difference *post hoc* test. In (c, d), asterisks indicate significant differences from wild type (Col‐0; *, *P* < 0.05; **, *P* < 0.01; ns, not significant), as determined by a two‐sided Student's *t*‐test. Values in (d) were normalized to the value in wild type (Col‐0) in week 2. (e) Histochemical staining (Bar, 1 mm) for and (f) quantification of β‐glucuronidase (GUS) activity in the shoot apex and young leaves of water (mock) or 20 μM dexamethasone (DEX)‐treated (for 2 d) 10‐d‐old LD‐grown *p35S:AHL15‐GR* plants expressing the miR156‐resistant *pSPL9:rSPL9‐GR*, *pSPL2:rSPL2‐GUS*, *pSPL9:rSPL9‐GUS*, *pSPL10:rSPL10‐GUS*, *pSPL11:rSPL11‐GUS*, *pSPL13:rSPL13‐GUS*, or *pSPL15:rSPL15‐GUS* reporter. In (e), the dotted circles mark the regions used for quantification in (f). Dots in (f) indicate the values of 10 biological replicates per plant line, the horizontal line and the number below this line indicate the mean, and error bars indicate the SEM. *, *P* < 0.05; **, *P* < 0.01; indicate a significant difference between mock‑ and DEX‐treated samples as determined by a two‐sided Student's *t*‐test. (g) Shape of the successive rosette leaves of 7‐wk‐old *p35S:MIM156* or *p35S:AHL15 p35S:MIM156* plants grown under SD conditions. For the rosette leaf shape of wild‐type (Col‐0) control plants we refer to Fig. 1(b). (h) The length : width ratio of the second, fifth, and seventh leaves or (i) the juvenile leaf number (leaves without abaxial trichomes) of 7‐wk‐old wild‐type, *p35S:MIM156*, *p35S:AHL15*, or *p35S:AHL15 p35S:MIM156* plants grown under SD conditions. A coloured dot indicates the individual measurement per plant (*n* = 15 biologically independent plants per line), the horizontal line and the number below this line indicates the mean and error bars indicate the SEM. Different letters indicate statistically significant differences (*P* < 0.01) as determined by a one‐way ANOVA with Tukey's honest significant difference *post hoc* test.

In Arabidopsis, VPC is mediated by a gradual decrease in *miR156*/*miR157* expression, which increases the expression of the *SPL* genes that are targets of these miRNAs. *SPL* genes, in turn, promote the adult developmental programme in the shoot apex, resulting in the production of adult instead of juvenile leaves and eventually in the initiation of flowering (Wu *et al*., 2009). One possible mode of action of AHL proteins might be that they suppress *SPL* expression by enhancing the *miR156*/*miR157* pathway. A qPCR comparison of the expression of *SPL* genes (*SPL2*, *SPL9*, *SPL10*, *SPL11*, *SPL13*, and *SPL15*) known to promote VPC (Xu *et al*., 2016) showed that the transcript levels of *SPL2*, *SPL9*, *SPL13*, and *SPL15* were significantly upregulated in shoots of 10‐d‑old or 3‐wk‐old *ahl15 ahl19 p35S:amiRAHL20* triple mutant and *ahl15/+ pAHL15:AHL15‐ΔG* plants compared with wild‐type seedlings under, respectively, LD or SD conditions (Fig. 3a–c). Under these conditions plantlets developed four and five leaves, respectively, which were juvenile based on their morphology. In the *ahl15 ahl19* double mutant, only *SPL9* and *SPL13* showed enhanced expression. These results suggest that AHL proteins, namely AHL15, ‑19, and ‑20, repress the expression of *SPL2*, *SPL9*, *SPL13*, and *SPL15* during VPC.

Not all *SPL* genes tested were simultaneously upregulated in the *ahl15/+ pAHL15:AHL15‐ΔG* mutant background, and this made us wonder whether AHLs repress *SPL* expression independent of the *miR156*/*miR157* pathway. Indeed, the *pmiR156A:GUS*, *pmiR156B:GUS*, and *pmiR156D:GUS* reporters (Yu *et al*., 2015) did not show a major change in expression upon AHL15 activation by DEX treatment in the *p35S:AHL15‐GR* background (Fig. S7). Also, qPCR analysis showed that miR156/157 levels were not significantly different in 2‑ or 3‐wk‐old wild‐type or *ahl15/+ pAHL15:AHL15‐ΔG* seedlings grown under juvenile‐phase‐prolonging SD conditions (Fig. 3d). In addition, when testing the expression of six miR156/miR157‐insensitive *pSPL:rSPL‐GUS* reporters (*rSPL2*, *rSPL9*, *rSPL10*, *rSPL11*, *rSPL13*, and *rSPL15*) (Xu *et al*., 2016) in the *p35S:AHL15‐GR* background, the expression of *rSPL2*, *rSPL9*, *rSPL13*, and *rSPL15* appeared to be downregulated by DEX treatment in the shoot apex, petioles, and leaves (Fig. 3e,f).

In plants overexpressing a target mimic of *miR156* (*p35S:MIM156*), *SPL* gene expression is enhanced (Franco‐Zorrilla *et al*., 2007), resulting in the accelerated appearance of adult leaves (Fig. 3g,h) and early flowering (Fig. S8a,b). *AHL15* overexpression negated the precocious appearance of adult vegetative traits (Fig. 3g,h) and early flowering (Fig. S8) of *p35S:MIM156* plants, bringing these traits back to or close to wild‐type levels, whereas the *MIM156* expression level was not affected by the *p35S:AHL15* construct (Fig. S9).

In the reverse experiment, where the *p35S:AHL15* construct was introduced into the *p35S:miR156* background having low SPL levels, we observed a remarkably additive effect on both the vegetative and the reproductive phase transition (Fig. S10a,b). qPCR analysis showed that *AHL15* and *miR156* overexpression levels were maintained in the *p35S:AHL15 p35S:miR156* background (Fig. S11a,b). Together, these results indicate that AHL15 and family members suppress the expression of specific *SPL* genes involved in VPC in an miR156/miR157‐independent manner.

### SPLs promote the vegetative phase change in part by repressing *AHL15* expression

**Fig. 4:**
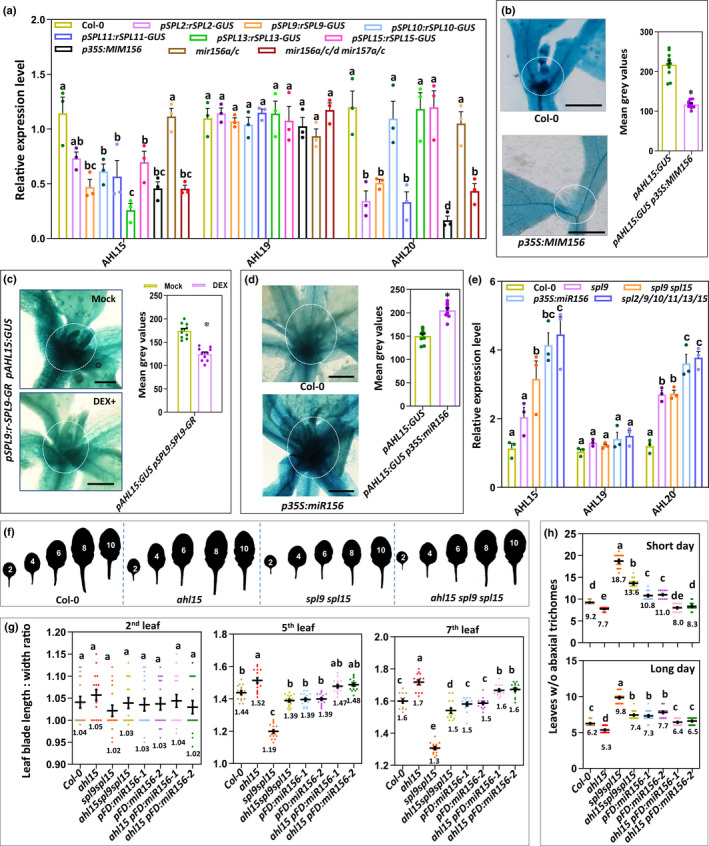
SQUAMOSA PROMOTOR BINDING PROTEIN‐LIKEs (SPLs) promote vegetative phase change by repressing *AT‐HOOK MOTIF NUCLEAR LOCALIZED 15* (*AHL15*) and *AHL20* expression in *Arabidopsis thaliana*. (a) Relative expression level of *AHL15*, *AHL19*, or *AHL20* in the shoot apex and young leaves of 10‐d‐old wild‐type (Col‐0), *pSPL2:rSPL2‐GUS*, *pSPL9:rSPL9‐GUS*, *pSPL10:rSPL10‐GUS*, *pSPL11:rSPL11‐GUS*, *pSPL13:rSPL13‐GUS*, *pSPL15:rSPL15‐GUS*, *p35S:MIM156*, or *mirR156a/b/d miR157a/c* seedlings grown under long day (LD) conditions. (b–d) Histochemical staining for (left) and quantification of (right) β‐glucuronidase (GUS) activity in the shoot apical meristem and leaf primordia of (b) 8‐d‐old *pAHL15:GUS* or *p35S:MIM156 pAHL15:GUS* seedlings, (c) 10‐d‐old water (mock) or 20 μM dexamethasone (DEX)‐treated *pAHL15:GUS p35S:SPL9‐GR* seedlings, or (d) 15‐d‐old *pAHL15:GUS* or *pAHL15:GUS p35S:miR156* seedlings grown under LD conditions. Bar, 1 mm. Dots in (b–d) indicate the values of 10 biological replicates per plant line, horizontal line and the number below this line indicate the mean, and error bars indicate the SEM. *, *P* < 0.01, indicates a significant difference with wild‐type background as determined by a two‐sided Student's *t*‐test. (e) Relative expression level of *AHL15*, *AHL19*, and *AHL20* in the shoot apex and young leaves of 14‐d‐old wild‐type, *spl9*, *spl9 spl15*, *p35S:miR156*, or *spl2/9/10/11/13/15* plants grown under LD conditions. In (a) and (e), dots indicate the values of three biological replicates per plant line, the bars indicate the mean, and error bars the SEM. Different letters indicate statistically significant differences (*P* < 0.05) as determined by a one‐way ANOVA with Tukey's honest significant difference *post hoc* test. (f) Shape of the successive rosette leaves of 7‐wk‐old wild‐type (Col‐0), *ahl15*, *spl9 spl15*, and *ahl15 spl9 spl15* plants grown under short day (SD) conditions. (g) The length : width ratio of the second, fifth, and seventh leaves of 7‐wk‐old wild‐type, *ahl15*, *spl9 spl15*, and *ahl15 spl9 spl15* plants grown under SD conditions. (h) The juvenile leaf number (leaves without abaxial trichomes) in wild‐type, *ahl15*, *spl9 spl15*, and *ahl15 spl9 spl15* plants grown under SD (top) or LD (bottom) conditions. Coloured dots in (g, h) indicate the individual measurement per plant (*n* = 15 biologically independent plants per line), the horizontal line and the number below this line indicate the mean, and error bars indicate the SEM. Different letters indicate statistically significant differences (*P* < 0.01) as determined by one‐way ANOVA with Tukey's honest significant difference *post hoc* test.

During VPC in 2‐wk‐old Arabidopsis seedlings, increasing SPL levels (Wang *et al*., 2009) coincided with downregulation of *AHL15*, *AHL19*, and *AHL20* (Fig. 2a–c). This led us to hypothesize that the SPL transcription factors are mediating downregulation of *AHL* gene expression. Based on this hypothesis, we expected *AHL* expression to be downregulated in Arabidopsis lines with elevated SPL levels (*pSPL:rSPL‐GUS* lines, *p35S:MIM156*, and *mirR156a/c/d mirR157a/c*) and to be upregulated in Arabidopsis lines with reduced SPL levels (*p35S:miR156* or *spl* loss‐of‐function mutants). qPCR analysis indeed showed that the expression of *AHL15* and *AHL20* was significantly reduced in 10‐d‐old Arabidopsis seedlings of lines with elevated SPL levels compared with wild‐type seedlings (Fig. 4a). In line with this result, expression of the *pAHL15:GUS* reporter was reduced in 1‐wk‐old seedlings overexpressing *MIM156* (Figs 4b, S12a), or in DEX‑ compared with mock‐treated *pSPL9:rSPL9‐GR* seedlings (Fig. 4c). By contrast, expression of the *pAHL15:GUS* reporter was enhanced in 2‐wk‐old seedlings by *miR156* overexpression (Figs 4d, S12b). qPCR analysis also showed enhanced expression of *AHL15* and *AHL20* in 2‐wk‐old *p35S:miR156*, *spl9spl15*, and *spl2/9/10/11/13/15* seedlings compared with wild‐type seedlings (Fig. 4e). Based on our hypothesis, the delayed VPC and flowering phenotypes of plants with reduced SPL levels (e.g. the *spl2/9/10/11/13/15* mutants and *p35S:miR156*) would be dependent on the elevated expression of functional *AHL* genes. The *ahl15* loss‐of‐function mutation in the *ahl15 spl9 spl15* triple mutant indeed partially rescued the delayed VPC phenotypes of the *spl9 spl15* double mutant. The length : width ratio of *ahl15 spl9 spl15* leaves was almost restored to wild‐type levels, and VPC of *ahl15 spl9 spl15* plants was significantly accelerated compared with that of *spl9 spl15* double‐mutant plants, both in SD and in LD conditions (Fig. 4f–h). Introduction of the *p35S:miR156* construct in the *ahl15* loss‐of‐function background also rescued the delay in VPC induced by *miR156* overexpression back to wild‐type levels (Fig. S13a,b), even in heterozygous *ahl15/+ p35S:miR156* plants. Since we could not exclude that the transfer DNA insertion in the *ahl15* mutant silenced the *p35S:miR156* construct (Fig. S13c; Daxinger *et al*., 2008), we placed the *miR156* gene under control of the *FD* promotor (*pFD:miR156*) and introduced the resulting construct into the *ahl15* mutant and wild‐type background. The delay in VPC caused by *FD*‐promotor‐controlled *miR156* expression in the wild‐type background was significantly reduced in the *ahl15* background both under SD and LD conditions (Fig. 4f–h). These results indicate that a functional *AHL15* gene is required in order to observe a delay in VPC in plants with reduced SPL levels. Together, the data confirm our hypothesis that the repression of *AHL*s in the shoot apex is mediated by SPLs, either indirectly or directly by binding of these transcription factors to the *AHL* regulatory regions.

In contrast to the VPC results presented thus far, the *ahl15* loss‐of‐function mutation had no or only a marginal effect on the delayed flowering of the *spl9 spl15* or *pFD:miR156* plants (Fig. S14). It should be noted, however, that *ahl15* single or *ahl15 ahl19* double‐mutant plants show a wild‐type flowering time and that only the *ahl15 ahl19 p35S:amiRAHL20* triple‐mutant or the *ahl15/+ pAHL15:AHL15‐ΔG*, *pAHL15:AHL15‐GUS*, or *ahl15/+ pAHL15:AHL15‐GUS* dominant‐negative mutant plants flower significantly earlier than the wild‐type does (Figs S2, S3; Karami *et al*., 2020). This indicates that not only *AHL15* but also other redundantly acting *AHL* genes, such as *AHL19* and *AHL20* (Fig. 4a), are the prime targets for repression by SPLs in the promotion of flowering.

### SPLs promote reproductive identity of axillary meristems by repressing *AHL15* expression

**Fig. 5:**
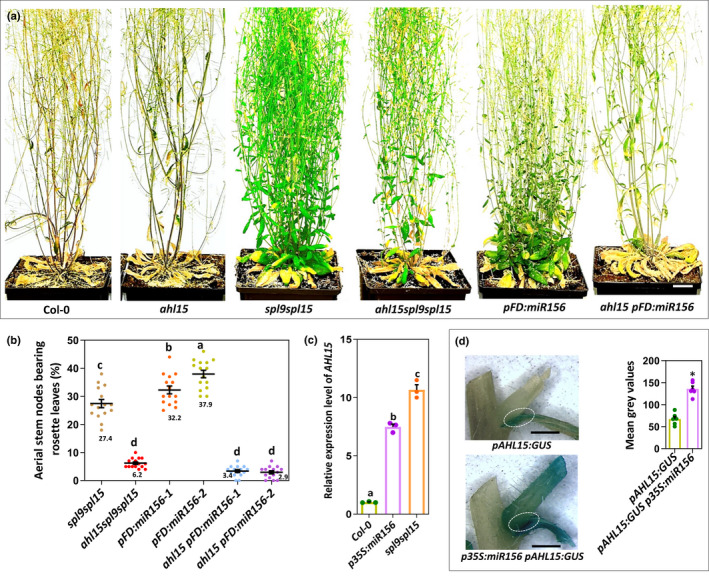
SQUAMOSA PROMOTOR BINDING PROTEIN‐LIKEs (SPLs) promote reproductive identity of axillary meristems by repressing *AT‐HOOK MOTIF NUCLEAR LOCALIZED 15* (*AHL15*) in *Arabidopsis thaliana*. (a) Phenotype of 3‐month‐old *spl9 spl15*, *ahl15 spl9 spl15*, *pFD:miR156*, or *pFD:miR156 ahl15* plants grown under long day (LD) conditions, and (b) percentage of aerial stem nodes on these plants bearing rosette leaves. Please note that wild‐type and *ahl15* stem nodes (not shown) do not form rosette leaves under these conditions. A coloured dot in (b) indicates the individual measurement per plant (*n* = 15 biologically independent plants per line), the horizontal line and the number below this line indicate the mean, and error bars indicate the SEM. Different letters indicate statistically significant differences (*P* < 0.01) as determined by one‐way ANOVA with Tukey's honest significant difference *post hoc* test. (c) The relative expression level of *AHL15* in aerial stem nodes of wild‐type (Col‐0), *p35S:miR156* or *spl9 spl15* plants 2 wk after flowering. Dots indicate the values of three biological replicates per plant line, horizontal line and the number below this line indicate the mean, and error bars indicate the SEM. Different letters indicate statistically significant differences (*P* < 0.01) as determined by a one‐way ANOVA with Tukey's honest significant difference *post hoc* test. (d) Histochemical staining for (left) and quantification of (right) β‐glucuronidase (GUS) activity in inflorescence nodes of 6‐wk‐old *pAHL15:GUS* and *pAHL15:GUS p35S:miR156* plants grown under LD conditions. In the graph, dots indicate the values of six biological replicates per plant line, the horizontal line and the number below this line indicate the mean, and error bars indicate the SEM. *, *P* < 0.01, indicates a significant difference with wild‐type background as determined by a two‐sided Student's *t*‐test. Bars: (a) 1 cm; (d) 50 mm.

Our previous results have shown that *AHL15* does play a central role in the developmental phase identity of AMs; that is, the vegetative to reproductive phase change (Karami *et al*., 2020). Overexpression of *AHL15* (*p35S:AHL15* or *pMYB:AHL15*) repressed this developmental switch, leading to prolonged vegetative activity of AMs and resulting in the formation of aerial rosette leaves from inflorescence nodes (Fig. S15a,b). Such aerial rosette leaves are not formed on wild‐type Arabidopsis (Col‐0) plants grown under LD conditions (Fig. S15a,b), but they can be induced by growing plants under SD conditions or by mutating the *SUPPRESSOR OF OVEREXPRESSION OF CONSTANS1* (*SOC1*) and *FRUITFULL* genes encoding transcription factors that suppress *AHL15* expression (Karami *et al*., 2020). Previously, we have demonstrated that the aerial rosette phenotype is dependent on a functional *AHL15* gene (Karami *et al*., 2020). Interestingly, the AMs in the axils of cauline leaves of *spl9 spl15* mutant or *p35S:miR156* (or *pFD:miR156*) overexpression plants also produced aerial rosette leaves (Fig. S15a,b), revealing an as yet unidentified role for SPLs in promoting the vegetative to reproductive transition of AMs. Introduction of *spl9 spl15* or *pFD:miR156* in the *ahl15* loss‐of‐function mutant background strongly reduced the aerial rosette leaf phenotype of both mutant lines (Fig. 5a,b), supporting our previous conclusion that *AHL15* function is important for this phenotype. As aerial rosette leaves are reproducibly absent in wild‐type and *ahl15* plants when grown under the same LD conditions (Fig. 5a), we did not take them along in this analysis. In line with the role of the SPL transcription factors as *AHL* repressors, *AHL15* expression was significantly increased in *spl9 spl15* and *p35S:miR156* AMs compared with wild‐type AMs (Fig. 5c,d). Based on these results, we concluded that *AHL15* acts downstream of the *SPL* genes and that repression of *AHL15* by SPLs in wild‐type plants promotes the reproductive identity of AMs, whereas under conditions where SPL activity is reduced (e.g. in *p35S:miR156* or *spl9 spl15* plants), elevated *AHL15* expression enhances the vegetative activity of AMs, resulting in the formation of aerial rosette leaves.

## Discussion

Plants during their lifetime progress through distinct consecutive developmental phases. What drives and regulates the transition from one developmental phase to another is a longstanding fundamental question in plant developmental biology. In Arabidopsis and several other plants, VPC and the reproductive phase change (flowering) have been shown to be driven by the ageing pathway. In this pathway, the SPL transcription factors promote phase transitions, and miR156 and miR157 repress these transitions by targeting the *SPL* transcripts (Poethig, 2013; Teotia & Tang, 2015). Previously, we have shown that Arabidopsis *AHL15* and its paralogues enhance plant longevity by delaying maturation of AMs (Karami *et al*., 2020). Here, we show that AHL15 and its close homologue AHL20, and possibly also AHL19, interfere with the ageing pathway by repressing *SPL2/9/13/15* gene expression in an miRNA‐independent manner and that, in turn, *AHL15* and *AHL20* expression is repressed through feedback regulation by the SPLs. In addition, we show that this reciprocal negative feedback loop between *AHL15/19/20* and *SPL2/9/13/15* genes not only regulates VPC and flowering but also AM maturation, and thus controls plant longevity and life history.

**Fig. 6:**
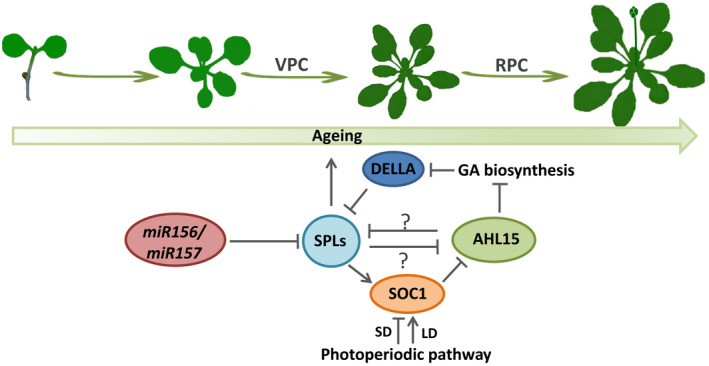
Proposed model for the role of AT‐HOOK MOTIF NUCLEAR LOCALIZED 15 (AHL15) and family members in the regulation of plant ageing. Following germination, high expression of microRNAs *miR156/miR157* and *AHL15* and family members keeps seedlings in the juvenile vegetative phase by suppressing the expression of the ageing‐promoting SQUAMOSA PROMOTOR BINDING PROTEIN‐LIKE (SPL) transcription factors. miR156/157 target the *SPL* messenger RNAs (Wang *et al*., 2009; Wu *et al*., 2009; He *et al*., 2018), and AHLs possibly bind directly to *SPL* loci (? indicates to be determined) or they may act indirectly by downregulating gibberellin (GA) biosynthesis, leading to accumulation of DELLA proteins that are known to repress *SPL* expression (Yu *et al*., 2012; Karami *et al*., 2020). When seedlings grow older, gradual downregulation of *miR156/157* elevates SPL abundance, which in turn promotes the juvenile to adult phase transition (or vegetative phase change, VPC) and the adult vegetative to reproductive phase transition (or reproductive phase change, RPC) by stimulating plant ageing in part by downregulating AHLs. SPLs possibly bind directly to the *AHL* upstream regions (? indicates to be determined) or promote the expression of SUPPRESSOR OF OVEREXPRESSION OF CONSTANS1 (SOC1), a negative regulator of the *AHL15* gene (Wang *et al*., 2009; Karami *et al*., 2020). The reciprocal negative feedback between AHLs and SPLs allows moderation of plant ageing by, for example, external signals such as short day (SD) and long day (LD) photoperiod through the floral integrator SOC1 (Huijser & Schmid, 2011). Arrows indicate activation, and blunted lines indicate repression.

Although the reproductive phase change is agronomically most important, VPC also has a strong impact on plant fitness and biomass, not in the least because it determines the balance between vegetative growth and reproductive development (Demura & Ye, 2010). Although the timing of VPC can be influenced by environmental factors such as photoperiod, light intensity, and temperature, this developmental switch is mainly regulated by endogenous genetic components (Poethig, 2013). Studies in Arabidopsis have revealed that the gradual decline in the expression of miR156/miR157 increases the abundance of the SPL transcription factors, which promote VPC (Poethig, 2013; Teotia & Tang, 2015). The change in leaf morphology during VPC is accompanied by a reduced expression of *AHL15* and close homologues in the SAM and young leaves, which is in line with our model that *AHL* genes are repressed by SPLs (Fig. 6).

Recently, it has been reported that these internal factors at the SAM maintain the juvenile phase during early shoot development, but that the SAM plays a relatively minor role in the regulation of leaf identity at later stages of VPC (Fouracre & Poethig, 2019). Our observations that *ahl* loss‐of‐function plants (*ahl15/+ pAHL15:AHL15‐ΔG*) immediately form adult leaves and that *p35S:AHL15* seedlings form multiple juvenile leaves confirm that AHLs belong to the internal factors in the shoot apex that maintain the juvenile phase in early shoot development. VPC was also strongly delayed when we overexpressed *AHL15* specifically in the SAM and young leaf primordia using the *FD* and *ANT* promoters, suggesting that AHL might also regulate VPC through lateral organ‐derived signals (Yang *et al*., 2011, 2013; Yu *et al*., 2013).

Our analysis showed that AHL15 represses the expression of *SPL* genes in an miR156/miR157‐independent manner. Previous studies have shown that the abundance of miR156 strongly declines (*c*. 90%) at the shoot apex of Arabidopsis seedlings within a 2 wk period, whereas the RNA level for most *SPL* genes remains low or increases only slightly during this period (Xu *et al*., 2016). Here, we show that the *SPL2*, *SPL9*, *SPL13*, and *SPL15* expression levels are significantly higher at the shoot apex of *ahl15* loss of function than in wild‐type plants, whereas the miR156/miR157 levels did not differ between wild‐type or *ahl15* loss‐of‐function mutant plants. This indicates that the maintained repression of *SPL* expression during early plant development is mediated by AHL15 independent of miR156/157. How AHL15 represses *SPL* genes remains unknown. AHL15 might directly bind to the *SPL* loci. However, preliminary yeast one‐hybrid assays did not provide an indication for direct binding of AHL15 to the *SPL* promoter regions (data not shown). Recently, we have shown that *AHL15* overexpression suppresses the biosynthesis of the plant hormone gibberellin (GA; Karami *et al*., 2020). Since DELLA proteins, the degradation targets of GA signalling, have been shown to repress *SPL* expression (Yu *et al*., 2012), AHL15 may repress *SPL*s indirectly by reducing GA biosynthesis and thus stabilizing the DELLA proteins (Fig. 6).

Previously, it has been shown that SPLs promote trichome development on the abaxial side of leaves partially by repressing the AP2‐like transcription factors TOE1 and TOE2 (Wu *et al*., 2009; Wang *et al*., 2019; Xu *et al*., 2019). How SPLs promote the other adult leaf traits, such as leaf elongation and leaf serration, was not known until now. In this study, we show that the promotion of adult traits, including leaf elongation and trichomes on the abaxial side of leaves, is in part mediated by the repression of *AHL* gene expression by SPLs.

We have recently published that AHL15 globally reorganizes the chromatin configuration (Karami *et al*., 2021). Therefore, AHL15 may control the ageing pathway by inducing changes in higher‐order chromatin organization that lead to repression of *SPL* genes, GA_3_ biosynthesis, and other pathways. In animals, the contribution of higher‐order chromatin organization to ageing processes has been well documented (Moraes, 2014; Chandra & Kirschner, 2016). However, the actual involvement of higher‐order chromatin organization in the juvenile‐to‐adult transition in plants remains to be investigated.

In conclusion, based on our findings, we propose a model in which both miR156/157 and AHL15/20 proteins independently slow down plant ageing by repressing *SPL2/9/13/15* expression: miR156/157 by targeting the *SPL* mRNAs (Wang *et al*., 2009; Wu *et al*., 2009; He *et al*., 2018) and AHL15/19/20 possibly by binding directly to *SPL* loci or indirectly through the DELLA proteins by downregulating biosynthesis of the ageing‐promoting GA (Yu *et al*., 2012; Karami *et al*., 2020) (Fig. 6). In turn, SPLs reciprocally repress *AHL* expression possibly by binding directly to the *AHL* upstream regions, or indirectly by promoting the expression of SOC1, a negative regulator of the *AHL15* gene (Wang *et al*., 2009; Karami *et al*., 2020) (Fig. 6). The floral integrator SOC1 (Huijser & Schmid, 2011) connects *AHL15* to the photoperiodic pathway, which explains why, under SD conditions, *AHL15* expression in the Arabidopsis SAM and AMs is enhanced, leading to delayed VPC and the production of aerial rosette leaves. Our findings place AHL15 and its close homologue AHL20, and possibly also AHL19, together with SPLs in a reciprocal regulatory feedback loop that allows modulation of the ageing pathway in plants by both internal (e.g. miRNA156/157) and external (e.g. photoperiod) signals.

## Author contributions

AR, OK and RO conceived the project, designed the experiments, and analysed and interpreted the results. AR performed most of the experiments. SB and OK contributed to some of the experiments. OK and RO supervised the project. AR, OK and RO wrote the manuscript. All authors read and commented on versions of the manuscript.

## Supporting information


**Fig. S1** Preparation of shoot apex and young leaf samples from young *Arabidopsis thaliana* plants for RNA isolation.
**Fig. S2** Expression of a dominant negative AHL15‐GUS fusion protein leads to precocious development of adult traits and early flowering in *Arabidopsis thaliana*.
**Fig. S3** *Arabidopsis thaliana AHL15* and close homologues redundantly regulate flowering time.
**Fig. S4** Extreme delay of vegetative phase change and flowering by heterologous expression of *Arabidopsis thaliana AHL15* in *Nicotiana tabacum*.
**Fig. S5** Shoot apical meristem‑ or young leaf‐specific *AHL15* overexpression delays flowering time in *Arabidopsis thaliana*.
**Fig. S6** Tissue‐specific *AHL15* overexpression in *Arabidopsis thaliana*.
**Fig. S7** *AHL15* does not affect the expression of *miR156A*, ‑*B*, or ‑*D* in *Arabidopsis thaliana*.
**Fig. S8** AHL15 and SPLs antagonistically control flowering time in *Arabidopsis thaliana*.
**Fig. S9** Overexpression of the *mimic miR156* (*p35S:MIM156*) is not altered by *p35S:AHL15* in *Arabidopsis thaliana*.
**Fig. S10** AHL15 and SPLs synergistically control vegetative phase change and flowering time in *Arabidopsis thaliana*.
**Fig. S11** *AHL15* and *miR156* are overexpressed in *Arabidopsis thaliana p35S:miR156 p35S:AHL15* plants.
**Fig. S12** Overexpression of the *mimic miR156* (*p35S:MIM156*) or *miR156* (*p35S:miR156*) in the *Arabidopsis thaliana pAHL15:GUS* background.
**Fig. S13** The rescue of *miR156* overexpression phenotypes by *ahl15* loss of function in *Arabidopsis thaliana* is most likely caused by silencing of the *p35S:miR156* construct.
**Fig. S14** Delay of flowering by *spl* loss of function in *Arabidopsis thaliana* is largely *AHL15* independent.
**Fig. S15** Aerial rosette leaves in *Arabidopsis thaliana* by reduced *SPL* expression or *AHL15* overexpression.
**Table S1** Gene IDs and primers used for cloning, genotyping, and quantitative PCR.
Please note: Wiley Blackwell are not responsible for the content or functionality of any Supporting Information supplied by the authors. Any queries (other than missing material) should be directed to the *New Phytologist* Central Office.

## Data Availability

The raw experimental data and the plant lines described are available upon request. The Arabidopsis AGI locus identifier for each gene described is provided in the Table S1.
