# Supplementary material for: miR156‐independent repression of the ageing pathway by longevity‐promoting AHL proteins in Arabidopsis
Source: New Phytol. 2022 Jun 26;235(6):2424–38. doi: 10.1111/nph.18292 (PMC9540020; doi:10.1111/nph.18292)
Supplement: Supplementary file 1 — Fig. S1 Preparation of shoot apex and young leaf samples from young Arabidopsis thaliana plants for RNA isolation. Fig. S2 Expression of a dominant negative AHL15‐GUS fusion protein leads to precocious development of adult traits and early flowering in Arabidopsis thaliana. Fig. S3 Arabidopsis thaliana AHL15 and close homologues redundantly regulate flowering time. Fig. S4 Extreme delay of vegetative phase change and flowering by heterologous expression of Arabidopsis thaliana AHL15 in Nicotiana tabacum. Fig. S5 Shoot apical meristem‑ or young leaf‐specific AHL15 overexpression delays flowering time in Arabidopsis thaliana. Fig. S6 Tissue‐specific AHL15 overexpression in Arabidopsis thaliana. Fig. S7 AHL15 does not affect the expression of miR156A, ‑B, or ‑D in Arabidopsis thaliana. Fig. S8 AHL15 and SPLs antagonistically control flowering time in Arabidopsis thaliana. Fig. S9 Overexpression of the mimic miR156 (p35S:MIM156) is not altered by p35S:AHL15 in Arabidopsis thaliana. Fig. S10 AHL15 and SPLs synergistically control vegetative phase change and flowering time in Arabidopsis thaliana. Fig. S11 AHL15 and miR156 are overexpressed in Arabidopsis thaliana p35S:miR156 p35S:AHL15 plants. Fig. S12 Overexpression of the mimic miR156 (p35S:MIM156) or miR156 (p35S:miR156) in the Arabidopsis thaliana pAHL15:GUS background. Fig. S13 The rescue of miR156 overexpression phenotypes by ahl15 loss of function in Arabidopsis thaliana is most likely caused by silencing of the p35S:miR156 construct. Fig. S14 Delay of flowering by spl loss of function in Arabidopsis thaliana is largely AHL15 independent. Fig. S15 Aerial rosette leaves in Arabidopsis thaliana by reduced SPL expression or AHL15 overexpression. Table S1 Gene IDs and primers used for cloning, genotyping, and quantitative PCR. Please note: Wiley Blackwell are not responsible for the content or functionality of any Supporting Information supplied by the authors. Any queries (other than missing material) should be direc [file NPH-235-2424-s001.pdf]

New Phytologist

***New Phytologist* Supporting Information**

Article title: **miR156-independent repression of the ageing pathway by longevity-promoting AHL proteins in Arabidopsis**

Authors: **Arezoo Rahimi, Omid Karami, Salma Balazadeh and Remko Offringa**

Article acceptance date: 25 May 2022

The following Supporting Information is available for this article:

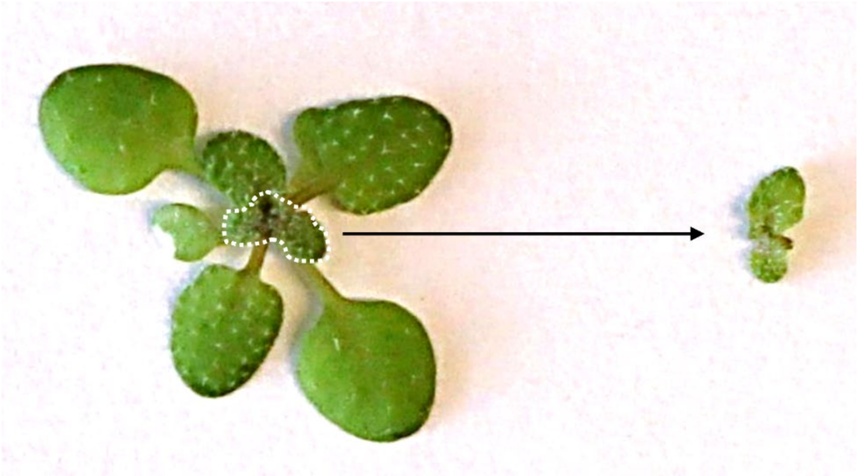

Fig. S1 Preparation of shoot apex and young leaf samples from young *Arabidopsis thaliana* plants for RNA isolation.

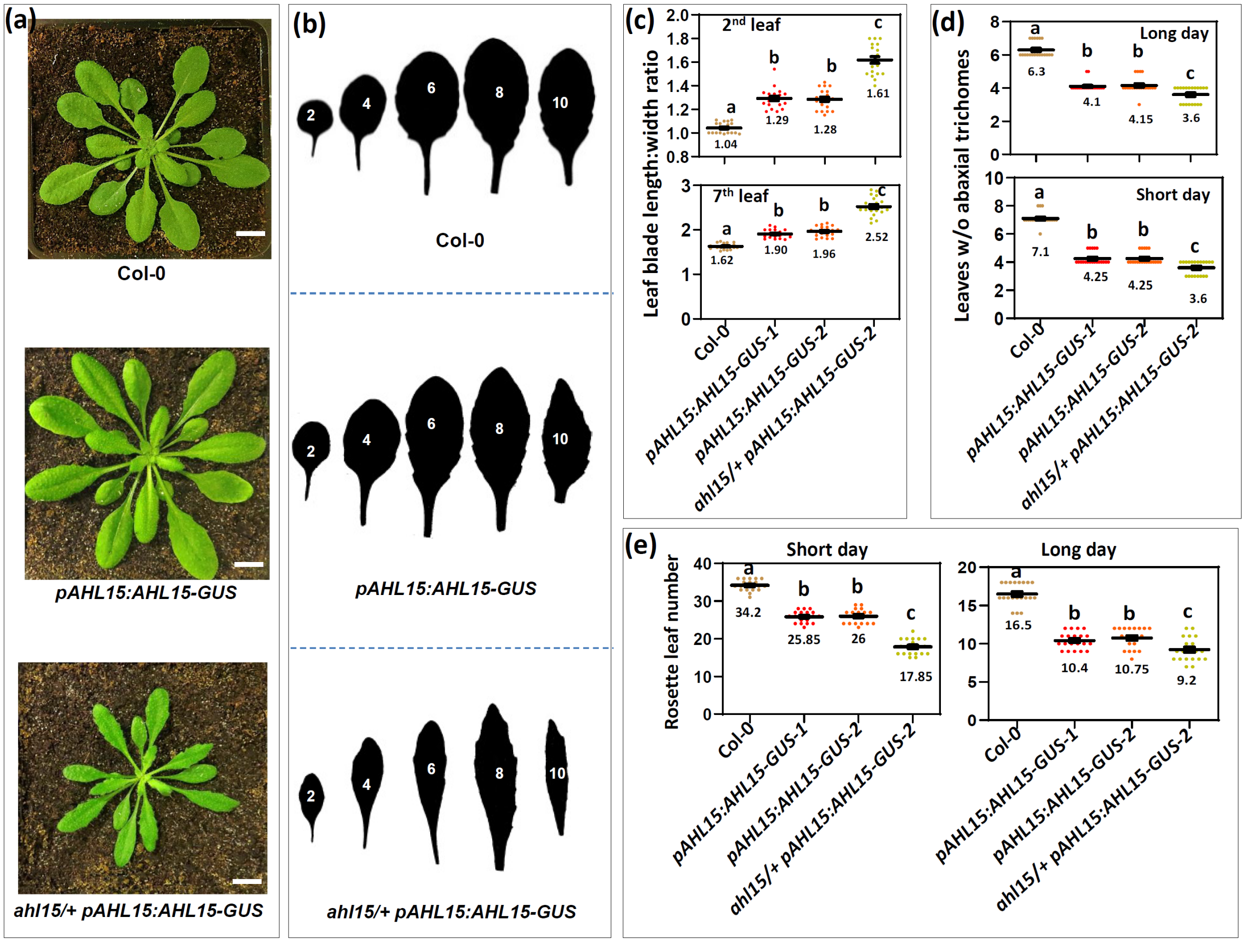

Fig. S2 Expression of a dominant negative AHL15-GUS fusion protein leads to precocious development of adult traits and early flowering in *Arabidopsis thaliana*. (**a**) The rosette phenotype of 6-week-old wild-type (Col-0), *pAHL15:AHL15-GUS* and *ahl15/+ pAHL15:AHL15-GUS* plants grown in short day (SD) conditions. Size bars indicate 2 cm. (**b**) Shape of the successive rosette leaves of 7-week-old of wild-type (Col-0), *pAHL15:AHL15-GUS* and *ahl15/+ pAHL15:AHL15-GUS* plants grown in SD conditions. (**c**) The length:width ratio of the 2nd and 7th leaf of 7-week-old wild-type, *pAHL15:AHL15-GUS* and *ahl15/+ pAHL15:AHL15-GUS* plants grown under SD conditions. (**d**) The juvenile leaf number (leaves without abaxial trichomes) in wild-type (Col-0), *pAHL15:AHL15-GUS* and *ahl15/+ pAHL15:AHL15-GUS* plants grown under SD and lopng day (LD) conditions. (**e**) The number of rosette leaves produced until flowering in wild-type (Col-0), *pAHL15:AHL15-GUS* and *ahl15/+ pAHL15:AHL15-GUS* plants grown under LD and SD conditions. (**c-e**) A coloured dot indicates the individual measurement per plant (n = 15

biologically independent plants per line), the horizontal line and the number below this line indicates the mean and error bars indicate the standard error of the mean. Different letters indicate statistically significant differences ($P < 0.01$) as determined by a one-way ANOVA with Tukey's honest significant difference post hoc test.

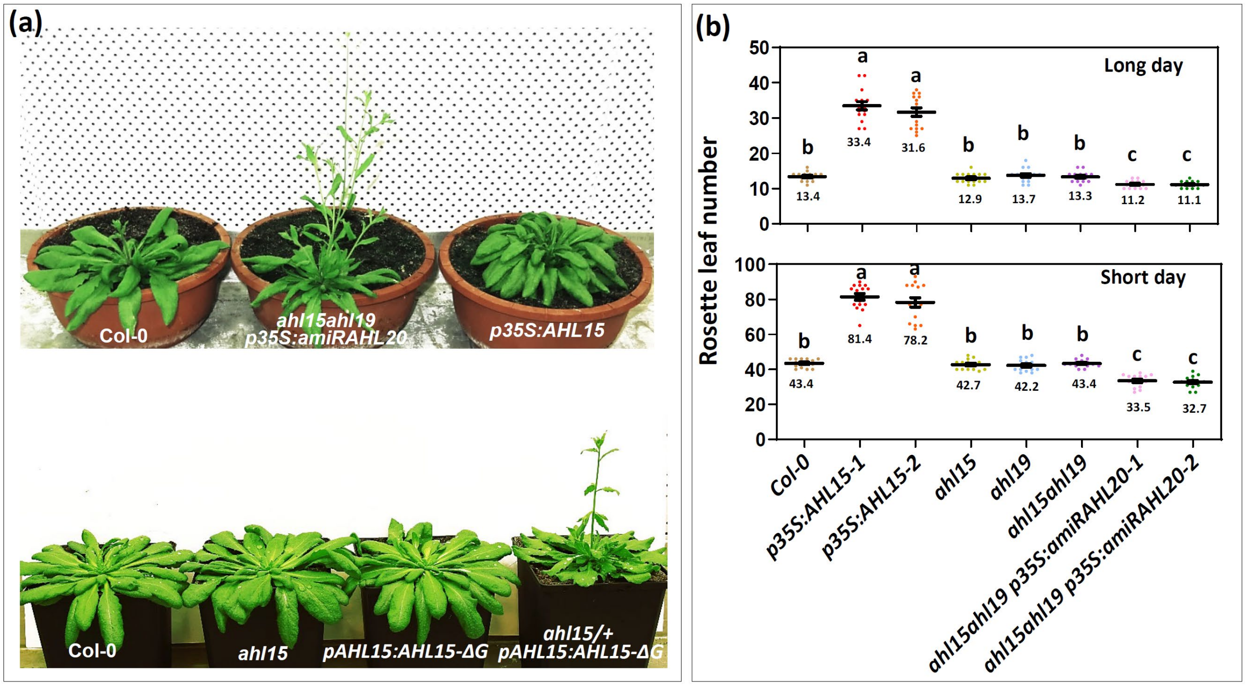

Fig. S3 *Arabidopsis thaliana AHL15* and close homologs redundantly regulate flowering time. (**a**) Phenotype of a 8-week-old wild-type (Col-0), *ahl15ahl19 p35S:amiRAHL20* or *p35S:AHL15* plant grown under short day (SD, 10-hour photoperiod) conditions (top) or of a 10-week-old wild-type (Col-0), *alh15*, *pAHL15:AHL15-ΔG* or *ahl15/+ pAHL15:AHL15-ΔG* plant grown under SD (8-hour photoperiod) conditions (bottom). (**b**) The number of rosette leaves produced until flowering by wild-type (Col-0), *p35S:AHL15, ahl15, ahl19, ahl15ahl19,* or *ahl15ahl19 p35S:amiRAHL20* plants grown under long day or SD conditions. A coloured dot indicates the individual measurement per plant (n = 15 biologically independent plants per line), the horizontal line and the number below this line indicates the mean and error bars indicate the s.e.m.. Different letters indicate statistically significant differences (P < 0.01) as determined by a one-way ANOVA with Tukey's honest significant difference post hoc test.

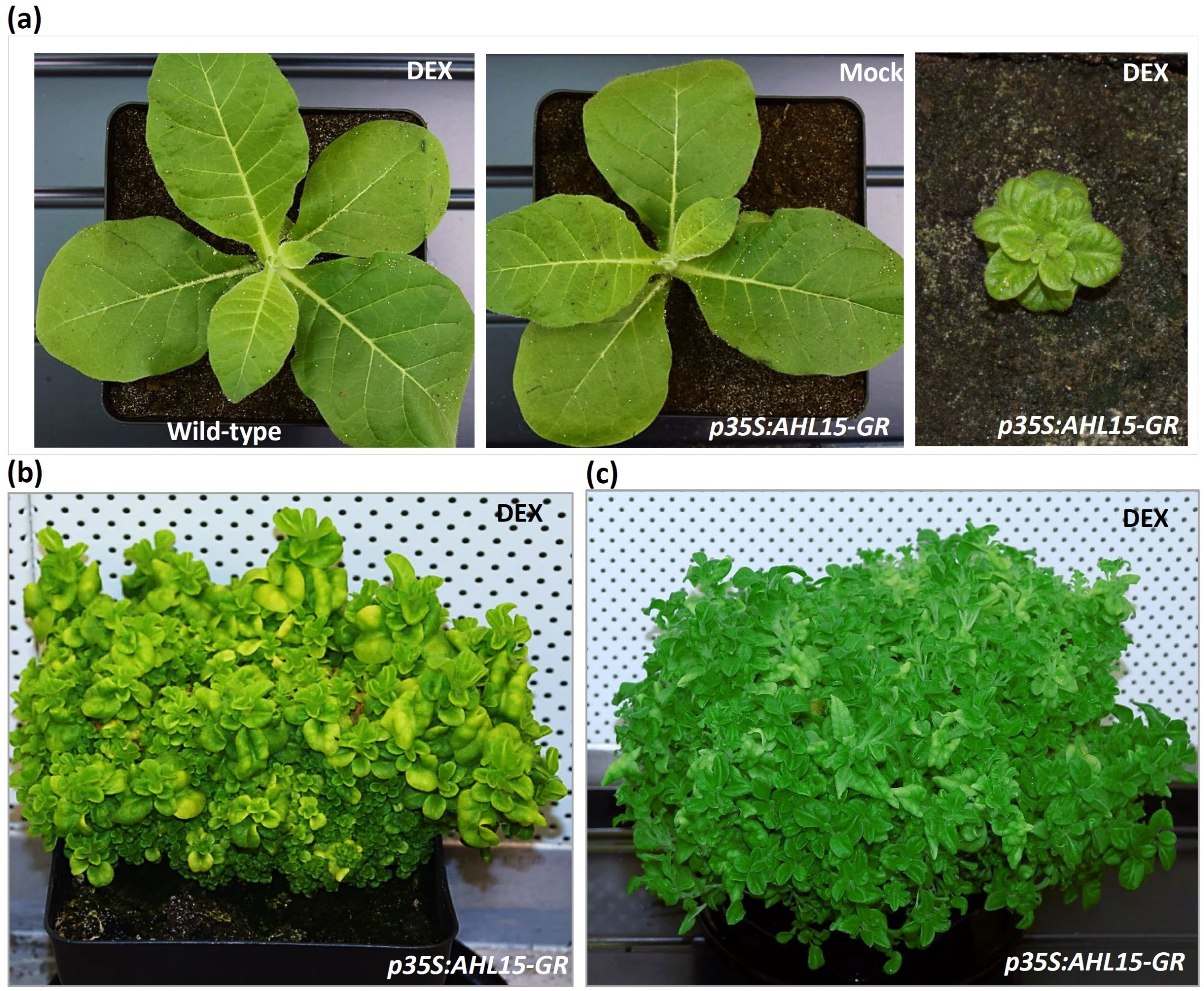

Fig. S4 Extreme delay of vegetative phase change and flowering by heterologous expression of *Arabidopsis thaliana AHL15* in *Nicotiana tabacum*. (**a**) Shoot morphology of a one-month-old wild-type (left) or *p35S:AHL15-GR* (middle and right) plant sprayed with water (Mock, middle) or sprayed with 20 µM dexamethason (DEX, left and right). (**b**, **c**) A six-month-old (**b**) or a one-year-old *p35S:AHL15-GR* (**c**) plant sprayed every week with 20 µM DEX.

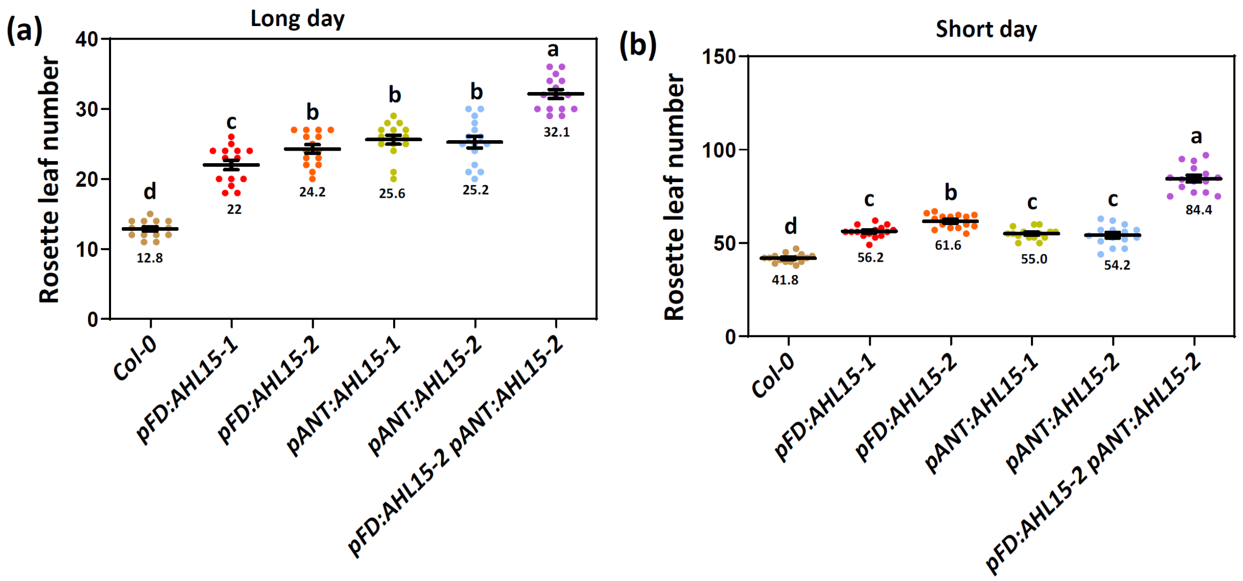

Fig. S5 Shoot apical meristem- or young leaf-specific *AHL15* overexpression delays flowering time in *Arabidopsis thaliana*. (**a**, **b**) The number of rosette leaves produced until flowering by wild-type (Col-0), *pFD:AHL15*, *pANT:AHL15* and *pFD:AHL15 pANT:AHL15* plants grown under long day (**a**) or short day (**b**) conditions. A coloured dot indicates the individual measurement per plant (n = 15 biologically independent plants per line), the horizontal line and the number below this line indicates the mean and error bars indicate the standard error of the mean. Different letters indicate statistically significant differences ($P < 0.01$) as determined by a one-way ANOVA with Tukey's honest significant difference post hoc test.

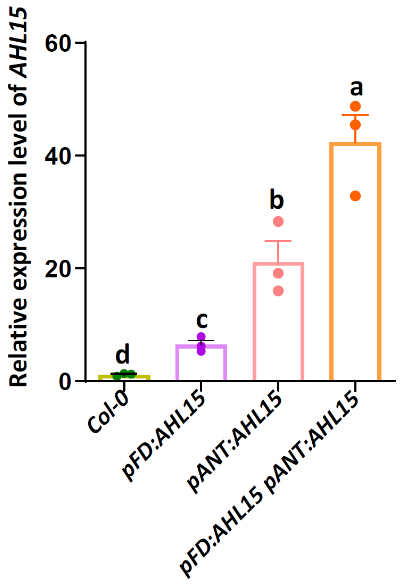

Fig. S6 Tissue-specific *AHL15* overexpression in *Arabidopsis thaliana*. The relative expression level of *AHL15* in the shoot apex and young leaves of 10-day-old wild-type, *pFD:AHL15*, *pANT:AHL15* and *pFD:AHL15 pANT:AHL15* plants grown under long day conditions. Dots indicate the values of three biological replicates per plant line, the bars indicates the mean and error bars indicate the standard error of the mean.

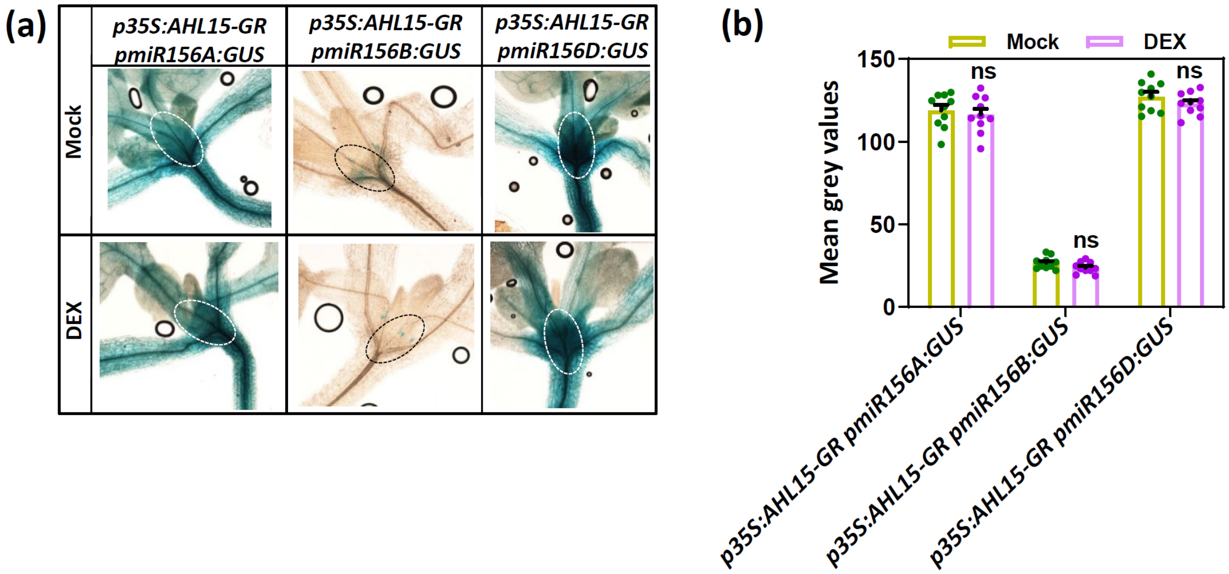

Fig. S7 *AHL15* does not affect the expression of *miR156A, B or D* in *Arabidopsis thaliana*. (**a**,**b**) Histochemical staining for (**a**) and quantification of (**b**) tissue-specific β-glucuronidase (GUS) activity in 2-week-old seedlings transgenic for *p35S:AHL15-GR* and *pmiR156A:GUS, pmiR156B:GUS* or *pmiR156C:GUS* expression following treatment with water (Mock, top) or 20 µM dexamethason (DEX, bottom). White or black dotted line indicates region used for quantification of the mean gray values in **b**. In **b,** dots indicate the values of eight biological replicates per plant line, horizontal line and the number below this line indicate the mean and error bars indicate the standard error of the mean. ns = not significantly different from mock treatment as determined by a two-sided Student's *t*-test.

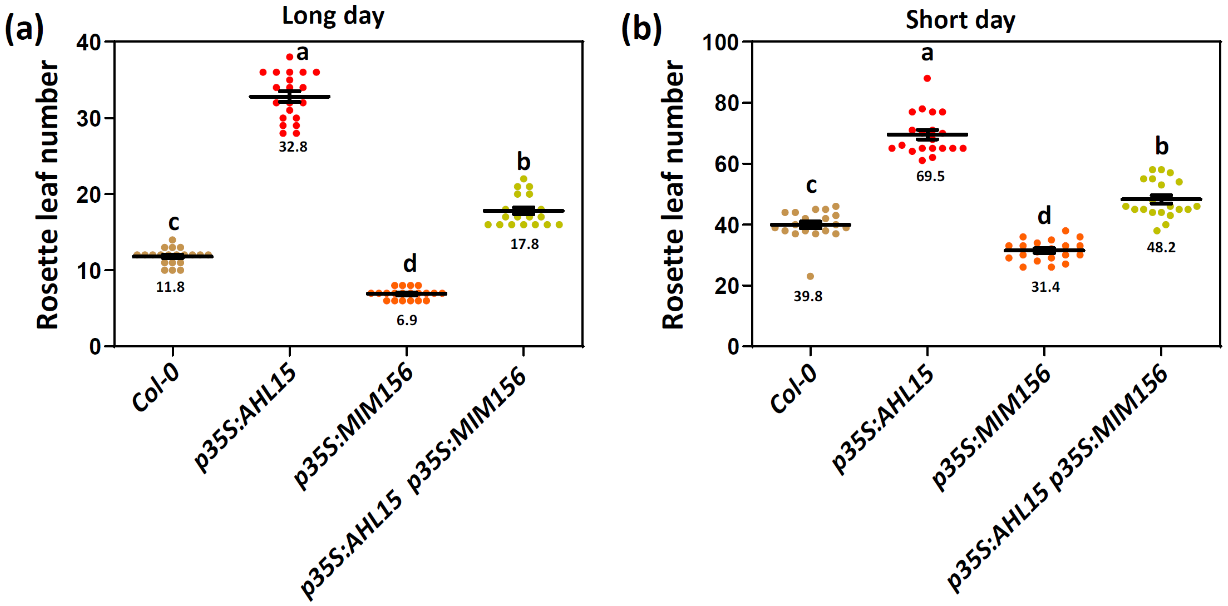

Fig. S8 AHL15 and SPLs antagonistically control flowering time in *Arabidopsis thaliana*. (**a**, **b**) The number of rosette leaves produced until flowering by wild-type (Col-0), *p35S:AHL15*, *p35S:MIM156* and *p35S:AHL15 p35S:MIM156* plants grown under long day (**a**) or short day (**b**) conditions. A coloured dot indicates the individual measurement per plant (n = 15 biologically independent plants per line), the horizontal line and the number below this line indicates the mean and error bars indicate the standard error of the mean. Different letters indicate statistically significant differences (P < 0.01) as determined by a one-way ANOVA with Tukey's honest significant difference post hoc test.

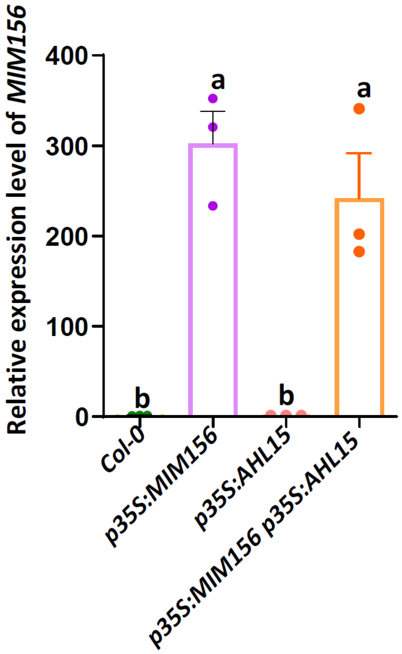

Fig. S9 Overexpression of the *mimic miR156* (*p35S:MIM156*) is not altered by *p35S:AHL15* in *Arabidopsis thaliana.* The relative expression level of *MIM156* in the shoot apex and young leaves of 10-day-old wild-type, *p35S:AHL15*, *p35S:MIM156* and *p35S:AHL15 p35S:MIM156* plants grown under long day conditions. Dots indicate the values of three biological replicates per plant line, the bars indicates the mean and error bars indicate the standard error of the meain. Different letters indicate statistically significant differences ($P < 0.01$) as determined by a one-way ANOVA with Tukey’s honest significant difference post hoc test

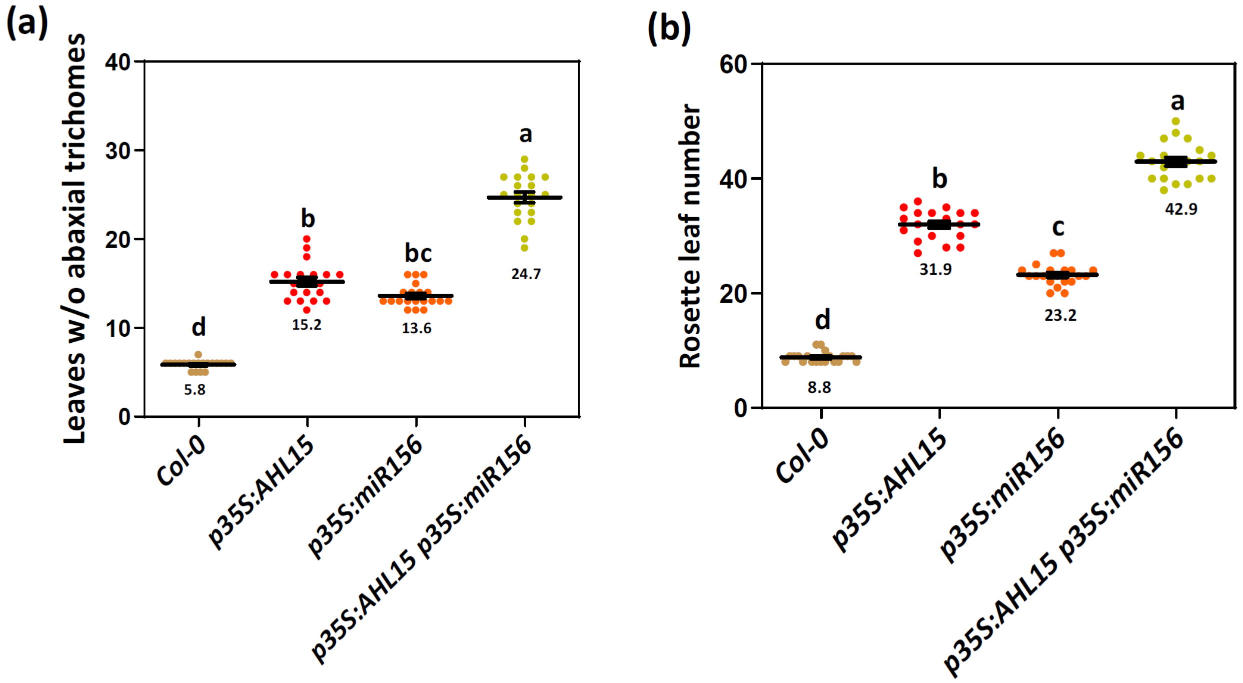

Fig. S10 AHL15 and SPLs synergistically control vegetative phase change and flowering time in *Arabidopsis thaliana*. (**a**, **b**) The juvenile leaf number (leaves without abaxial trichomes) (**a**) and the number of rosette leaves produced until flowering (**b**) in wild-type, *p35S:AHL15*, *p35S:miR156* and *p35S:AHL15 p35S:miR156* plants grown under long day conditions. A coloured dot indicates the individual measurement per plant (n = 15 biologically independent plants per line), the horizontal line and the number below this line indicates the mean and error bars indicate the standard error of the mean. Different letters indicate statistically significant differences (P < 0.01) as determined by a one-way ANOVA with Tukey's honest significant difference post hoc test.

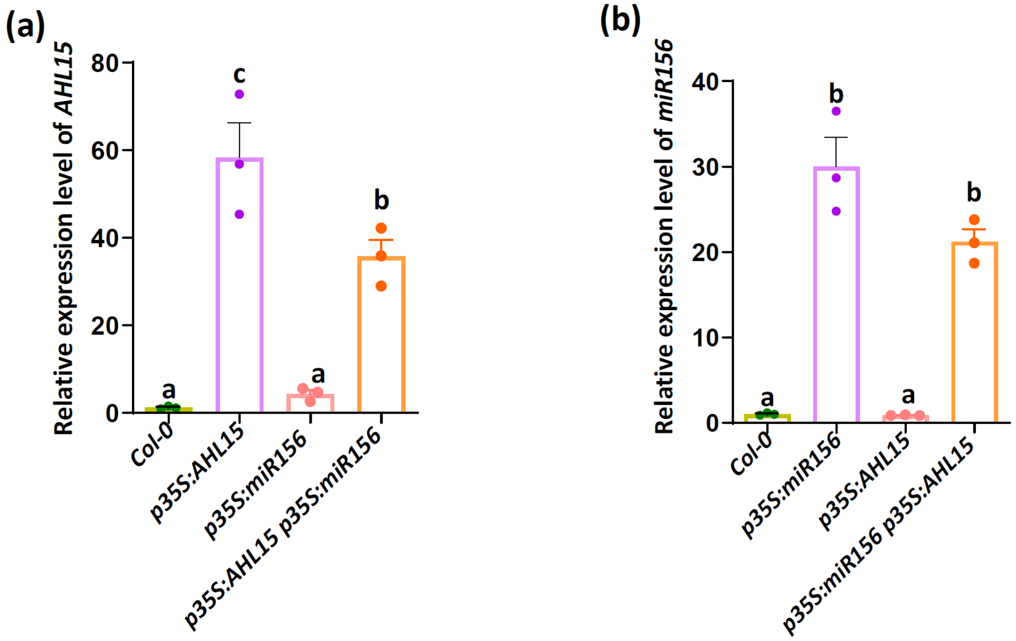

Fig. S11 *AHL15* and *miR156* are overexpressed in *Arabidopsis thaliana p35S:miR156 p35S:AHL15* plants. (**a**) The relative expression level of *AHL15* in the shoot apex and young leaves of 10-day-old wild-type, *p35S:AHL15*, *p35S:miR156* and *p35S:AHL15 p35S:miR156* plants grown under long day (LD) conditions. (**b**) The relative expression level of *miR156* in the shoot apex and young leaves of 10-day-old wild-type, *p35S:AHL15*, *p35S:miR156* and *p35S:AHL15 p35S:miR156* plants grown under LD conditions. In **a** and **b**, dots indicate the values of three biological replicates per plant line, the bars indicates the mean and error bars indicate the standard error of the mean. Different letters indicate statistically significant differences ($P < 0.01$) as determined by a one-way ANOVA with Tukey's honest significant difference post hoc test

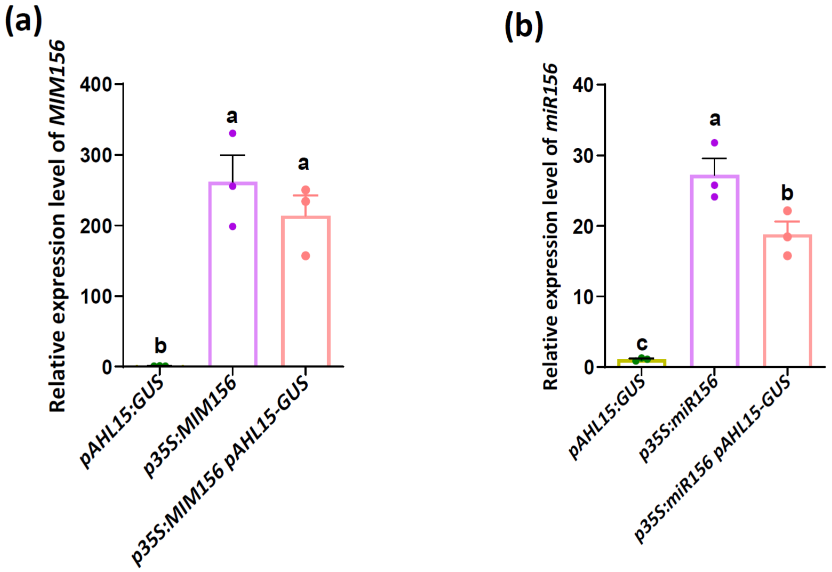

Fig. S12 Overexpression of the *mimic miR156* (*p35S:MIM156*) or *miR156* (*p35S:miR156*) in the *Arabidopsis thaliana pAHL15:GUS* background. (**a**) The relative expression level of *MIM156* in the shoot apex and young leaves of 10-day-old *pAHL15:GUS*, *p35S:MIM156* and *pAHL15:GUS p35S:MIM156* plants grown under long day (LD) conditions. (**b**) The relative expression level of *miR156* in the shoot apex and young leaves of 10-day-old *pAHL15:GUS*, *p35S:miR156* and *pAHL15:GUS p35S:miR156* plants grown under LD conditions. In **a** and **b**, dots indicate the values of three biological replicates per plant line, the bars indicates the mean and error bars indicate the standard error of the mean. Different letters indicate statistically significant differences ($P < 0.01$) as determined by a one-way ANOVA with Tukey's honest significant difference post hoc test.

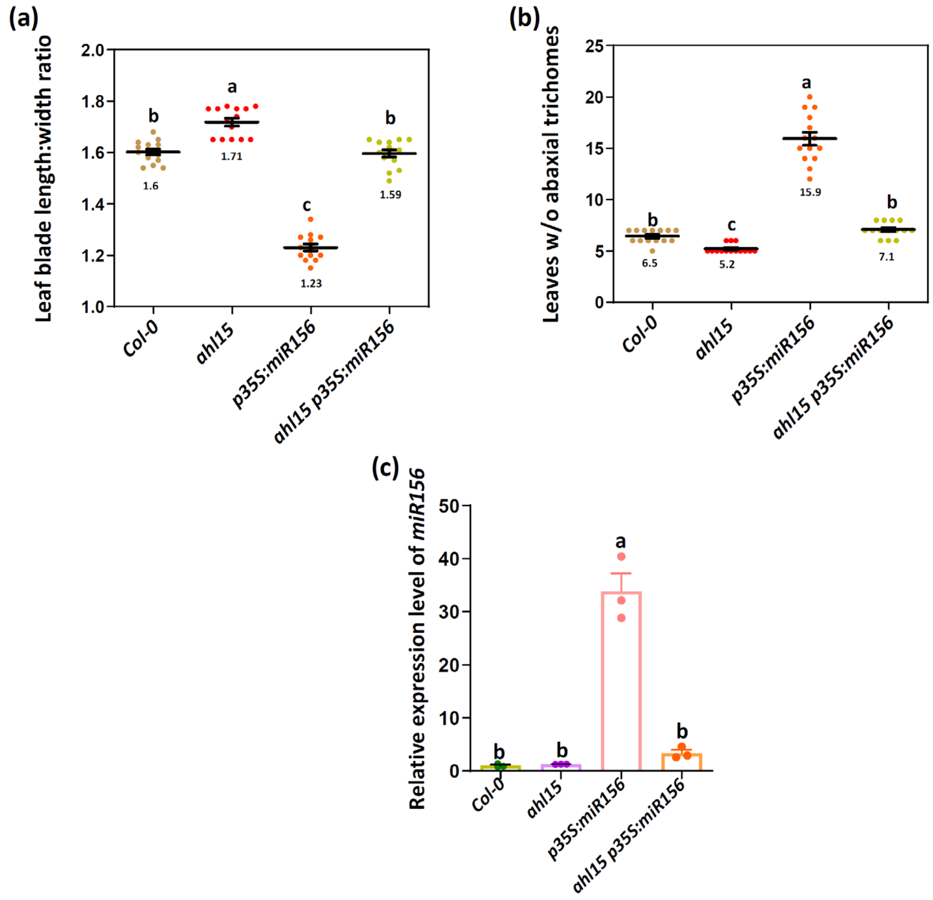

Fig. S13 The rescue of *miR156* overexpression phenotypes by *ahl15* loss-of-function in *Arabidopsis thaliana* is most likely caused by silencing of the *p35S:miR156* construct. (**a**) The length:width ratio of 7th leaf of 7-week-old wild-type (Col-0), *ahl15, p35S:miR156, ahl15, p35S:miR156* plants grown under short day (SD) conditions. (**b**) The juvenile leaf number (leaves without abaxial trichomes) in wild-type (Col-0), *ahl15, p35S:miR156, ahl15, p35S:miR156* plants grown under SD conditions. In **a** and **b**, a coloured dot indicates the individual measurement per plant (n = 15 biologically independent plants per line), the horizontal line and the number below this line indicates the mean and error bars indicate the standard error of the mean. Different letters indicate statistically significant differences (P < 0.01) as determined by a one-way ANOVA with Tukey's honest significant difference post hoc test. (**c**) The relative expression level of *miR156* in the shoot apex and young leaves of 10-day-old wild-type, *ahl15, p35S:miR156, ahl15, p35S:miR156* plants grown under SD conditions. Dots indicate the values

of three biological replicates per plant line, the bars indicates the mean and error bars indicate the standard error of the mean. Different letters indicate statistically significant differences ($P < 0.01$) as determined by a one-way ANOVA with Tukey's honest significant difference post hoc test.

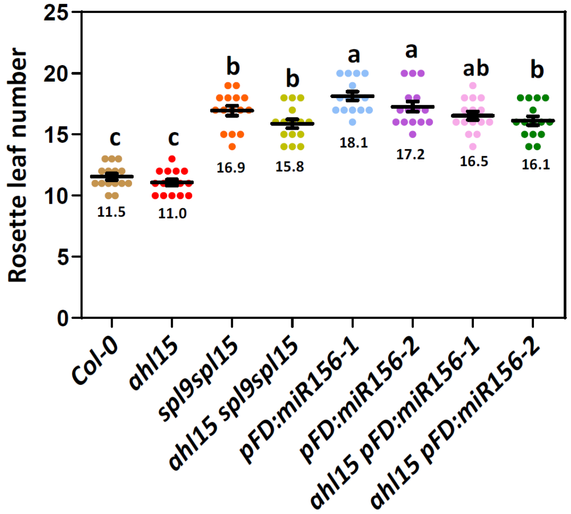

Fig. S14 Delay of flowering by *spl* loss-of-function in *Arabidopsis thaliana* is largely *AHL15*-independent. The number of rosette leaves produced until flowering by wild-type (Col-0), *ahl15*, *spl9spl15*, *ahl15spl9spl15, pFD:miR156*, *ahl15 pFD:miR156* plants grown under long day conditions. A coloured dot indicates the individual measurement per plant (n = 15 biologically independent plants per line), the horizontal line and the number below this line indicates the mean and error bars indicate the standard error of the mean. Different letters indicate statistically significant differences (P < 0.01) as determined by a one-way ANOVA with Tukey's honest significant difference post hoc test.

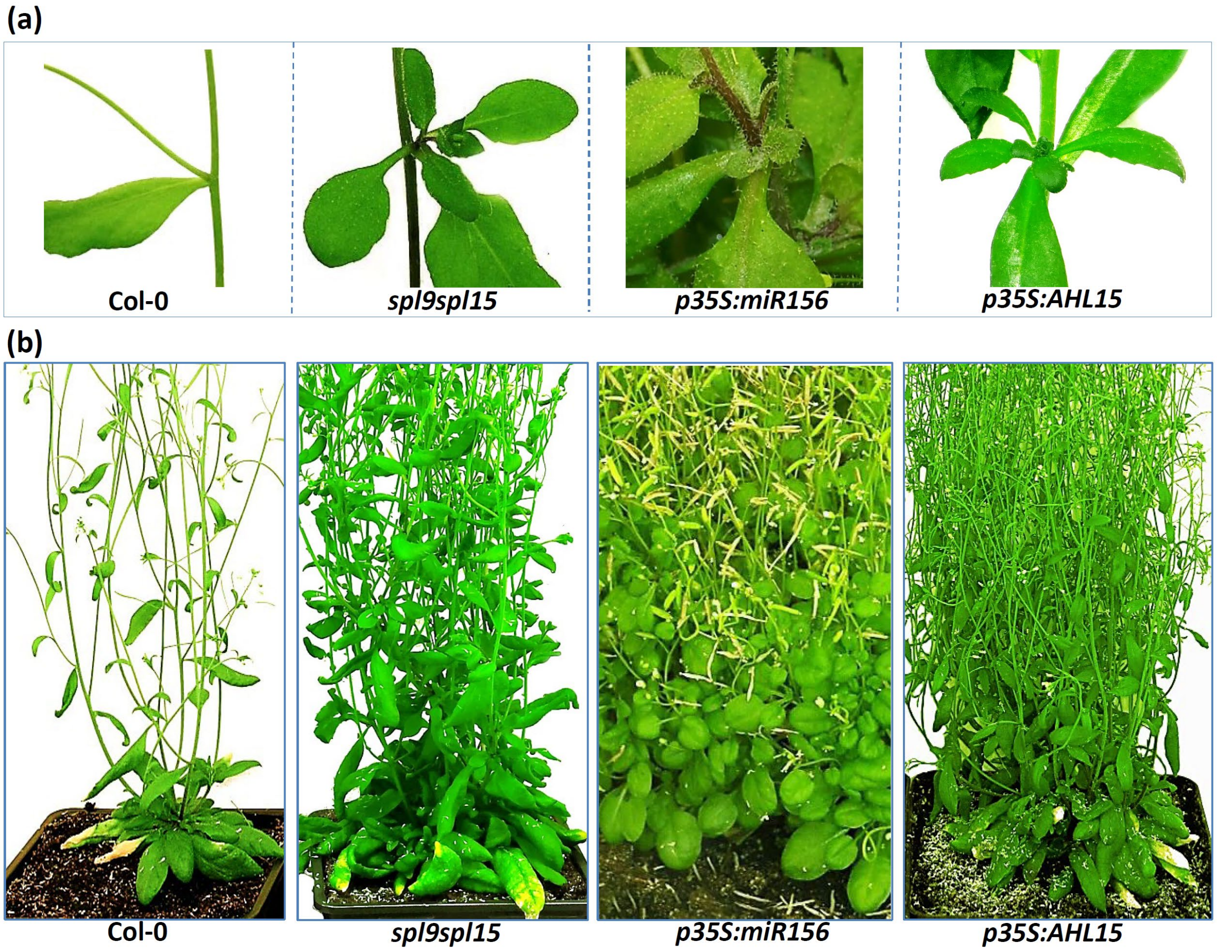

Fig. S15 Aerial rosette leaves in *Arabidopsis thaliana* by reduced *SPL* expression or *AHL15* overexpression. (**a**,**b**) A detail of the first node of an inflorescence (**a**) and the lower shoot phenotype (**b**) of flowering wild-type (Col-0), *spl9spl15*, *p35S:miR156* or *p35S:AHL15* plants grown under long day conditions.

New Phytologist

Table S1. Gene IDs and primers used for cloning, genotyping and qPCR

Gene ID	Primer name*	Sequence (5' to 3')	Purpose
See below	*KpnI -miR156-F*	CAGGTACCGTAAGACACGTGTAGAAATC	*pFD:miR156*
	miR156-R-SpeI	GGGTGAAGCACATTAGATACTAGTACC	
AT4G35900	*pFD-F*	GGGGACAAGTTTGTACAAAAAAGCAGGCTGGCCCTCTCTACTTGATTTAG	*pFD:AHL15*
	pFD-R	GGGGACCACTTTGTACAAGAAAGCTGGGTATGGAAAAGAGAACAGAAGTGAAC	
AT4G37750	*pANT-F*	GGGGACAAGTTTGTACAAAAAAGCAGGCTCATACGCTTGGAAGGGATAAG	*pANT:AHL15*
	pANT-R	GGGGACCACTTTGTACAAGAAAGCTGGGTAGGTTTCTTTTTTTGGTTTCTGC	
AT2G25095	miR156-RT-PCR	GTCGTATCCAGTGCAGGGTCCGAGGTATTCGCACTGGATACGACGTGCTCA	*qRT-PCR miR156*
AT4G30972			
AT4G31877			
AT5G10945			
AT5G11977			
AT5G26147			
AT2G19425			
AT5G55835			
AT1G66783	miR157-RT-PCR	GTCGTATCCAGTGCAGGGTCCGAGGTATTCGCACTGGATACGACGTGCTCT	*qRT-PCR miR157*
AT1G66795			
AT3G18217			
AT1G48742			
	At snoR101-F	CTTCACAGGTAAGTTCGCTTG	*qRT-PCR miR*
	At snoR101-R	AGCATCAGCAGACCAGTAGTT	
AT2G42200	spl9-F	TGGTTCCTCCACTGAGTCATC	*spl9* genotyping
	spl9-R	GCTCATTATGACCAGCGAGTC	
AT3G57920	spl15-F	TGTTGGTGTCTGAAGTTGCTG	*spl15* genotyping
	spl15-R	TCCACCGAGTCTTCTTCACTC	
AT3G55560	SALK_040729-F	GTCGGAGAGCCATCAACACCA	*ahl15* genotyping
	SALK_040729-R	CGACGACCCGTAGACCCGGATC	
AT3G55560	qAHL15-F	AAGAGCAGCCGCTTCAACTA	*qRT-PCR AHL15*
	qAHL15-R	TGTTGAGCCATTTGATGACC	
AT3G04570	qAHL19-F	CTCTAACGCGACTTACGAGAGATT	*qRT-PCR AHL19*
	qAHL19-R	ATATTATACACCGGAAGTCCTTGGT	
AT4G14465	qAHL20-F	CAAGGCAGGTTTGAAATCTTATCT	*qRT-PCR AHL20*
	qAHL20-R	TAGCGTTAGAGAAAGTAGCAGCAA	
See above	qmiR157-F	GCGGCGGTTGACAGAAGATAG	*qRT-PCR miR157*
for miR157	qmiR157-R	GTGCAGGGTCCGAGGT	
See above	qmiR156-F	GCGGCGGTGACAGAAGAGAGT	*qRT-PCR miR156*
for miR156	qmiR156-R	GTGCAGGGTCCGAGGT	
AT5G12250	qβ-TUBULIN-6-F	TGGGAACTCTGCTCATATCT	*qRT-PCR β-TUBULIN-6*
	qβ-TUBULIN-6-R	GAAAGGAATGAG GTTCACTG	
AT5G43270	qSPL2-F	TTTCCGATACCGAGCACAATAG	*qRT-PCR SPL2*
	qSPL2-R	TACGGGTTGGAGGTTGCTTGAGG	
AT2G42200	qSPL9-F	AATTGGCGACTCAAACTGTG	*qRT-PCR SPL9*
	qSPL9-R	CTGAAGAAGCTCGCCATGTA	
AT1G27370	qSPL10-F	CAGACAAAGGTGTGGGAGAATGCTC	*qRT-PCR SPL10*
	qSPL10-R	TAGGGAAAGTGCCAAATATTGGCG	
AT1G27360	qSPL11-F	AGTCCAAGTTTCAACTTCATGGCG	*qRT-PCR SPL11*
	qSPL11-R	GAACAGAGTAGAGAAAATGGCTGC	
AT5G50570	qSPL13-F	GCTCGAGAACCGCATCGTT	*qRT-PCR SPL13*
	qSPL13-R	CCCGTAAAAAACTGTCTCAACTGCT	
AT3G57920	qSPL15-F	TGAATGTTTTATCACATGGAAGCTC	*qRT-PCR SPL15*
	qSPL15-R	TCATCGAGTCGAAACCAGAAGATG	
AT3G09922	qIPS1-F	CAAACACCACAAAAACAAAAGA	*qRT-PCR IPS1*
	qIPS1-R	AAGAGGAATTCACTATAAAGAG	

*, F: forward; R: reverse; IPS1: primer pair used for MIM156 detection
